# Identifying phenotypic and genetic traits for assessing pathogenic potential and biocontrol capacity in *Burkholderia sensu lato* strains

**DOI:** 10.1093/ismejo/wrag081

**Published:** 2026-04-10

**Authors:** Kirsty Agnoli, Anugraha Mathew, Stefano Gualdi, Sarah Paszti, Lionel Moulin, Annette Vergunst, Peter Mergaert, Leo Eberl

**Affiliations:** Department of Plant and Microbial Biology, University of Zurich, 8008 Zurich, Switzerland; Department of Plant and Microbial Biology, University of Zurich, 8008 Zurich, Switzerland; Department of Plant and Microbial Biology, University of Zurich, 8008 Zurich, Switzerland; Department of Plant and Microbial Biology, University of Zurich, 8008 Zurich, Switzerland; Plant Health Institute of Montpellier (PHIM), IRD, CIRAD, INRAE, Institut Agro, Univ Montpellier, 34394 Montpellier, France; VBIC, Univ Montpellier, INSERM, 30908 Nîmes, France; Université Paris-Saclay, CEA, CNRS, Institute for Integrative Biology of the Cell, 91198 Gif-sur-Yvette, France; Department of Plant and Microbial Biology, University of Zurich, 8008 Zurich, Switzerland

**Keywords:** pathogenicity, biocontrol, *Burkholderia s.l*, *Galleria mellonella*, antifungal activity, siderophores, proteases, oxalotrophy

## Abstract

In the 1990s, several *Burkholderia* strains were registered as biocontrol agents but later withdrawn after opportunistic infections were reported. Phylogenetic revisions now separate the group into *Burkholderia sensu stricto* (*s.s.*), including environmental and clinical strains with elevated pathogenic potential, and several newly established genera, largely presumed harmless. We analysed 76 *Burkholderia sensu lato* (*s.l.*; taxa formerly classified as *Burkholderia*) for pathogenic potential in a *Galleria mellonella* model, *in vitro* biocontrol activity, and phenotypic traits linked to virulence or environmental fitness. Proteolytic activity and siderophore production manifested in strains with higher pathogenic potential, whereas oxalate utilization and other environmental traits correlated with lower pathogenic potential. Whereas most pathogenic strains belonged to *B. s.s.*, some *Paraburkholderia* isolates also exhibited elevated pathogenic potential. Transfer of the ornibactin biosynthetic cluster from a clinical *Burkholderia* strain to environmental *Paraburkholderia sacchari* increased pathogenic potential without affecting biocontrol capacity or persistence, illustrating the fine line between safe and hazardous strains. Collectively, our results identify novel phenotypic traits and genetic markers that enable improved, strain-level evaluation of pathogenic potential and biocontrol capacity, supporting the rational selection or engineering of *Burkholderia s.l.* strains for safe agricultural applications.

## Graphical abstract

**Graphical Abstract:**
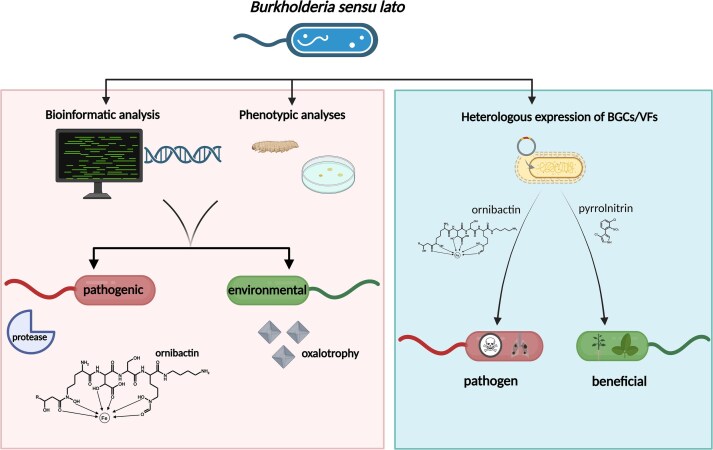

(Created in BioRender, license https://BioRender.com/p308dtj)

## Introduction

When the genus *Burkholderia* was defined in 1992, it consisted of only seven species [1], all of which were associated with disease: *Burkholderia pseudomallei* and *B. mallei* are primary pathogens of animals and humans [2, 3]; *B. caryophylli* (now *Trinickia caryophylli*) and *B. gladioli* are plant pathogens, and *B. cepacia* was originally described as the causative agent of sour skin rot of onion [4–6]. The remaining two species, the plant pathogen *B. solanacearum* and the opportunistic human pathogen *B. pickettii*, were later transferred to the genus *Ralstonia* [7]*.* In the following years, the number of *Burkholderia* species increased rapidly for two main reasons: (i) the emergence of various *B. cepacia*–like species as opportunistic pathogens causing severe infections in cystic fibrosis (CF) and immunocompromised patients [8–10] and (ii) the identification of species that promote plant growth and health or are capable of degrading recalcitrant pollutants [11–17]. These developments were underpinned by advances in sequencing technologies and refined taxonomic methods. A number of *Burkholderia* strains were noted for their potential for agricultural applications, particularly in controlling phytopathogenic fungi. Several strains were, at the time, registered in the USA and commercialized as biopesticides under the brand names Deny, Blue Circle, and Intercept. However, phylogenetic analysis revealed that the strains on which these products were based could not be reliably distinguished from CF isolates, and evidence emerged that strains isolated in the clinic originated from natural reservoirs, most likely the rhizosphere of plants [18]. As a consequence, the products were withdrawn from the market and, in 1999, the US Environmental Protection Agency (EPA) placed a moratorium on the registration of products containing potentially harmful *Burkholderia* species [especially members of the so-called *B. cepacia* complex (Bcc); see below] [16, 19, 20]. As a result, the genus *Burkholderia* has become a showcase illustrating the potential health risks associated with the use of microorganisms in commercial applications.

Since the moratorium was initiated, numerous *Burkholderia* species have been described from diverse natural environments that have rarely, if ever, been associated with infections or clinical settings. Many of these species promote plant growth, protect plants from pathogens, or enhance plant stress tolerance [21–23]. As reports indicating risks to human health were largely lacking, these organisms were considered suitable candidates for application. This prompted further investigation, ultimately leading to the division of the genus into two groups: *Paraburkholderia,* containing the supposedly harmless environmental species, and the *Burkholderia sensu stricto* (*s.s.*), comprising clinical isolates, phytopathogenic species, and some environmental species with both high pathogenic potential and often excellent biocontrol capabilities [24]. In addition to the primary pathogens *B. mallei* and *B. pseudomallei*, the causative agents of glanders and melioidosis [3], respectively, and some important plant pathogens such as *B. glumae, T. caryophylli* and *B. gladioli,* the *Burkholderia s.s.* also includes the Bcc. This group comprises at least 20 closely related species, most of which can cause opportunistic infections, especially in CF and immunocompromised patients [12, 25]. Of particular clinical importance is the Bcc species *B. cenocepacia* [26, 27]*,* which has often been associated with poor prognosis in CF patients [28]. Virtually all Bcc species have also been isolated from the environment, often from soil samples or from the rhizosphere of plants, and many of these strains have been demonstrated to exhibit excellent biocontrol activities [13, 20, 29–31]. Indeed, the strains used in the commercial biopesticides mentioned above all belonged to the Bcc [13, 32]. More recently, the *Burkholderia sensu lato* (*s.l.*) group (meaning *Burkholderia* in the loose sense; the genera that were *‘Burkholderia’* before phylogenetic revision) underwent further reclassification, leading to the creation of additional genera*,* including *Caballeronia, Trinickia*, *Robbsia*, *Pararobbsia,* and *Mycetohabitans* [33–36]. From an applications perspective, members of the genus *Paraburkholderia* show particularly high potential. Some strains nodulate legumes and fix atmospheric nitrogen (the so-called β-rhizobia), solubilize inorganic phosphate, synthesize phytohormones, lower the level of plant ethylene via the enzyme aminocyclopropane-1-carboxylate deaminase, or degrade environmental contaminants [37–39]. Certain *Paraburkholderia* strains can also live endophytically within plant roots. A well-studied example is *Paraburkholderia phytofirmans* PsJN [40, 41], which colonizes a range of economically important crops, promotes growth, and enhances tolerance to abiotic stresses such as drought, salinity, and temperature extremes (4°C and 32°C) [21–23], as well as tolerance to plant pathogenic microorganisms [42–44].

The current separation of the *Burkholderia s.l.* strains with lower versus higher pathogenic potential is primarily based on phylogenetic arguments, which, in some cases, are supplemented by bioinformatic predictions of putative virulence factors and beneficial traits. However, this approach often lacks experimental validation [15, 16, 24, 45, 46]. Furthermore, it remains unclear whether all *Burkholderia s.s.* strains exhibiting biocontrol activities are truly associated with elevated pathogenic potential and how beneficial and pathogenic traits are distributed across the *Burkholderia s.l.* clade [12, 47–49].

In this study, we tested a panel of 76 strains representing the genera *Burkholderia*, *Paraburkholderia*, *Caballeronia*, *Trinickia*, and *Robbsia* for pathogenic potential using a *Galleria mellonella* infection model, as well as for biocontrol activity in *in vitro* antimicrobial assays. We also examined various phenotypic traits associated with pathogenic potential or biocontrol capacity. Bioinformatic analysis of the corresponding genomes enabled us to link observed phenotypes with genotypic factors, some of which showed predictive value for assessing the pathogenic risk and biocontrol potential of a strain. To further investigate the role of the siderophore ornibactin, which is found exclusively in members of the *Burkholderia s.s.*, we transferred its biosynthetic gene cluster from the clinical isolate *B. cenocepacia* H111 into the nonpathogenic environmental isolate *P. sacchari* LMG 19450 and tested the resulting transgenic strains for virulence in the *G. mellonella* model, biocontrol capacity, and environmental persistence.

## Materials and methods

### Strains, media and growth conditions

All strains, oligonucleotides and plasmids have been listed in Tables S1, S2 and S3, respectively. Strains were streaked on no-salt lysogeny broth (NSLB) plates (1% (w/v) tryptone, 0.5% (w/v) yeast extract, 1.5% agar (Conda)) and incubated at room temperature or 30°C. Overnight precultures were grown for the inoculation of each of the assays (NSLB, 30°C overnight). Recombinant *P. tuberum and P. sacchari s*trains were grown at 37**°**C in iron-free succinate (IFS) medium [50] for growth assays, and 50 μM bipyridyl was included as an iron chelator where indicated.

### Phenotypic assays

Antibacterial activity against the plant pathogen *Pectobacterium carotovorum* was assessed using an agar bilayer assay as described previously [51]. The production of siderophores was assessed using the Chrome Azurol S (CAS) assay with modifications as detailed (see Supplementary Methods) [52]. Swimming motility was assessed on Petri dishes containing swimming medium [0.2% w/v agar, 1% w/v tryptone, 0.5% w/v yeast extract (DIFCO)]. The diameter of the bacterial swimming zone was measured at 48 h. Proteolytic activity was assessed on skimmed milk agar plates as described previously [53]. *Galleria mellonella* larvae in the final larval stage were purchased from Reptile-Food GmbH, Zürich, Switzerland, and the *G. mellonella* infection assay was carried out as described [51], using an inoculum of ~1 × 10^6^ colony forming units (CFU) per larva. The CFU ml^−1^ for each inoculum was checked by dilution series plating. All phenotypic assays were carried out in triplicate.

### PCR-based screen for the presence of megaplasmid pC3

The origin of replication of megaplasmid pC3 is highly conserved within the Bcc, allowing its presence to be easily screened by polymerase chain reaction (PCR) amplification [53, 54]. The primers used were BccRepF and BccRepR, which amplified an 800 bp fragment of pC3.

### Heterologous expression of the pyrrolnitrin cluster

A shorter version of the moderately expressed constitutive BBa_J23109 promoter (Standard Registry of Biological Parts), which we have previously used for heterologous expression of target genes in *Burkholderia* spp. [55] was synthesized as two oligonucleotides (dsJ23109For2 and dsJ23109Rev2). These were annealed and cloned between the *KpnI* and *HindIII* sites of pBBR1MCS, resulting in plasmid pBBR1MCS-pJ23109. The *prn* cluster was amplified using Phusion taq polymerase (NEB) with primers prnBamFor and prnXbaRev, using *B. lata* 383 genomic DNA extracted using the Wizard Genomic DNA Purification Kit (Promega) as template, and cloned into pBBR1MCS-pJ23109 between its *BamHI* and *XbaI* sites. After sequence confirmation, the plasmid was transferred to *P. sacchari* LMG 19450 and *P. tuberum* LMG 21444 by triparental conjugation*.*

### Microcosm experiments

Soil for microcosm experiments was collected from an agricultural field in Aberdeen (kindly provided by Nejc Stopnisek), autoclaved three times (121°C, 15 psi, 15 min), air-dried for 7 days, and sieved to <2 mm and stored at 4°C until use. Its physicochemical properties are listed in Table S4. For microcosms, 10 g of soil was placed in Petri dishes and adjusted to 60% of its water-holding capacity (measured as described in [56]). Each dish was inoculated with 100 μl of a 10^8^ CFU ml^−1^ bacterial suspension, and the inoculum was spread evenly. Plates were covered with aluminium foil and incubated at 25°C. Soil moisture was monitored every 2 days and adjusted with deionized water as needed. Wild-type and mutant populations were enumerated every second day for 4 or 10 days, as indicated. Assays were carried out in triplicate.

### Extraction of dissolved and adsorbed siderophores from soil

Adsorbed and dissolved siderophores were extracted as previously described [57] with minor modifications. For dissolved siderophores, 1 g of air-dried soil was mixed with 10 ml Milli-Q water, shaken for 2 h, and filtered using 0.45 μm filters (Millipore, Switzerland). The filtrates were concentrated by rotary evaporation, and the residues were dissolved in 1 ml Milli-Q water. For adsorbed siderophores, 1 g soil was extracted with 10 ml methanol for 2 h, filtered (0.45 μm), and the methanol evaporated; residues were again dissolved in 1 ml Milli-Q water. The extracted fractions and the initial siderophore inoculum were spotted onto CAS plates to assess siderophore content. The extractions and assays were carried out in triplicate.

### *In silico* analyses

The genomes of the 40 sequenced panel strains were analysed for genes that might link genotype with phenotype using various bioinformatic pipelines, as detailed in the Supplementary Materials.

### Statistical analyses

The statistical analyses indicated in the text were carried out using the Python packages Scipy.stats v1.17.0 (Brown–Forsythe and Mann–Whitney tests) and Lifelines 0.30.0 (Gehen–Breslow–Wilcoxon test), or Graphpad Prism 10 (Fisher’s exact test, paired *t*-test).

## Results

### Strain panel used in this study

To investigate the distribution of beneficial and pathogenicity-associated traits among the *Burkholderia s.l.* with the aim of identifying functions with predictive value for a risk assessment, we assembled a panel of strains from the three largest genera of this clade. Furthermore, to gain broader coverage across the *Burkholderia s.l.,* representatives were added from other genera where they were available in our in-house laboratory collection, to give a panel of 76 strains, with 35 *Paraburkholderia*, 34 *Burkholderia s.s.,* 5 *Caballeronia,* and 1 each from *Trinickia* and *Robbsia* (Table 1, Fig. S1). The genomes of 40 strains were available to link phenotypic traits with genomic information. In a first step, we subjected the strain panel to phenotypic assays as detailed below.

**Table 1:** The *Burkholderia s.l.* strain panel used within this study.

	**Strain name used in this study** ^**a**^	**ABIP panel name**	**Other key synonyms**	**Isolation source**	**Ref**	**Genome accession** ^**b**^
*R. andropogonis*	ICMP 2807		LMG 2129	*Sorghum bicolor*	[ 104 ]	8 063 489 567
*C. cordobensis*	LMG 27620	ABIP 2341		Soil	[ 105 ]	2 724 679 457
*C. grimmiae*	LMG 27580	ABIP 2339	R27	Moss (*Grimmia montana*)	[ 106 ]	2 609 460 223
*C. insecticola*	RPE64	ABIP 2336		*Riptortus pedestris* gut (symbiont)	[ 107 ]	2 597 489 944
*C. sordidicola*	LMG 22029			Moss (*Grimmia montana*)	[ 108 ]	2 713 896 994
*C. zhejiangensis*	OP-1	ABIP 2340		Sludge from the wastewater treatment plant of a pesticide manufacturing plant	[ 109 ]	2 772 190 654
*T. caryophylli*	LMG 2155		Ballard 720	*Dianthus caryophyllus*		GCA_040314805
*B. gladioli pv. gladioli*	ATCC 10248	ABIP 49	LMG 2216	*Gladiolus* sp*.*		2 639 762 983
*B. plantarii*	ATCC 43733	ABIP 121	CFBP 3573 T, ICMP 9424 T, NCPPB 3590, LMG 9035, JCM 5492, ICMP 9424	*Oryza sativa*		2 687 453 568
*B. glumae*	LMG 10906	ABIP 123	ICMP 3729, Dye MD4, NCPPB 3674	*Oryza sativa*, grain		
*B. glumae*	NCPPB 3923	ABIP 452		*Oryza sativa*		
*B. glumae*	LMG 2196	ABIP 48	ATCC 33617	*Oryza sativa*, grain		2 648 501 169
*B. thailandensis*	E264		LMG 20219	Rice field soil		637 000 052
*B. cepacia*	ATCC 25416	ABIP 4		Onion (*Allium cepa*)		2 912 464 834
*B. cepacia*	ABIP 2356		F2-P2	*Oryza sativa* IR504	Busset *et al*. (unpublished)	
Bcc *cepacia-*like	ABIP 441	ABIP 441	P14-NS, BCEP441	*Oryza sativa*	[ 110 ]	GCF_914492715.1
Bcc *cepacia-*like	ABIP 2227	ABIP 2227	P3-1 PCAT	*Oryza sativa* IR504	Busset *et al*. (unpublished)	
Bcc *cepacia*-like	ABIP 2230	ABIP 2230	P4-7 PCAT	*Oryza sativa* IR504	Busset *et al*. (unpublished)	
Bcc *cepacia*-like	ABIP2232	ABIP2232	P2-1-2 PCAT	*Oryza sativa* Zonghua	Busset *et al*. (unpublished)	
Bcc *cepacia*-like	ABIP 438	ABIP 438	P9-NS	*Oryza sativa*	Busset *et al*. (unpublished)	
Bcc *cepacia-like*	ABIP 449	ABIP 449	P39-NS	*Oryza sativa*	Busset *et al*. (unpublished)	
*B. cenocepacia*	H111			CF lung		GCA_000236215.4
*B. orbicola*	ABIP 443	ABIP 443	P19-NS	*Oryza sativa*	Busset *et al*. (unpublished)	
*B. orbicola*	ABIP 444	ABIP 444	P21-NS	*Oryza sativa*	[110]	GCA_905184115
Bcc *latens-*like	ABIP 2472	ABIP 2472	G7-2Camb19	*Oryza sativa*	Busset *et al*. (unpublished)	GCF_965118155.1
*B. diffusa*	ABIP 447	ABIP 447	P34-NS, BDI447	*Oryza sativa*	Busset *et al*. (unpublished)	GCA_914492725.1
*B. vietnamiensis*	ABIP 2229	ABIP 2229	P3-3 PCAT	*Oryza sativa* IR504	Busset *et al*. (unpublished)	
*B. vietnamiensis*	ABIP 434	ABIP 434	P24-ST, BVIET434	*Oryza sativa*	Busset *et al*. (unpublished)	GCF_914484955.1
*B. vietnamiensis*	ABIP 436	ABIP 436	P28-ST	*Oryza sativa*	Busset *et al*. (unpublished)	
*B. vietnamiensis*	ABIP 440	ABIP 440	P13-NS	*Oryza sativa*	Busset *et al*. (unpublished)	
*B. vietnamiensis*	LMG 10929	ABIP 47		*Oryza sativa*, rhizosphere soil		2 636 415 515
*B. vietnamiensis*	ABIP 670	ABIP 670	43 101	*Oryza sativa*	Busset *et al*. (unpublished)	
*B. vietnamiensis*	ABIP 1335	ABIP 1335	BF-233-1-PCAT, BVI1335	*Oryza sativa*	Busset *et al*. (unpublished)	GCF_914484905.1
*B. vietnamiensis*	ABIP 2221	ABIP 2221	P1-1 PCAT	*Oryza sativa* Zonghua	Busset *et al*. (unpublished)	
*B. vietnamiensis*	ABIP 2300	ABIP 2300		*Oryza sativa*	Busset *et al*. (unpublished)	
Bcc *multivorans-*like	ABIP 2354	ABIP 2354	B9-P2	*Oryza sativa*	Busset *et al*. (unpublished)	
Bcc *multivorans-*like	ABIP 2226	ABIP 2226	P2-7 PCAT	*Oryza sativa*	Busset *et al*. (unpublished)	GCA_965217245.1
Bcc *multivorans-*like	ABIP 2352	ABIP 2352	A6-P2	*Oryza sativa*	Busset *et al*. (unpublished)	
Bcc *multivorans-*like	ABIP 2225	ABIP 2225	P2-6 PCAT	*Oryza sativa*	Busset *et al*. (unpublished)	GCA_965217235.1
Bcc *multivorans-*like	ABIP 2353	ABIP 2353	B8-P2	*Oryza sativa*	Busset *et al*. (unpublished)	
Bcc *multivorans-*like	ABIP 2355	ABIP 2355	C3-P2	*Oryza sativa*	Busset *et al*. (unpublished)	
*P. bryophila*	1S18		LMG 23644	Moss (*Sphagnum rubellum*)		GCA_003269035
*P. megapolitana*	A10		CCUG 53007	Moss (*Aulacomnium palustre*)		
*P. megapolitana*	LMG 23650			Moss (*Aulacomnium palustre*)		
*P. phenazinium*	S18			Sphagnum moss		SRR14887065
*P. caribensis*	ABIP 1334	ABIP 1334	BF-232-1-PCAT, PCAR1334	*Oryza sativa*	[111]	GCF_914484885.1
*P. caribensis*	LMG 18531	ABIP 2642	MWAP-64, DSM 13236	Vertisol soil		GCA_002902945
*P. caribensis*	ABIP 462	ABIP 462	N26-ST	*Oryza sativa*	Busset *et al*. (unpublished)	
*P. caribensis*	ABIP 477	ABIP 477	N57-NS	*Oryza sativa*	Busset *et al*. (unpublished)	
*P. caribensis*	ABIP 478	ABIP 478	N60-ST	*Oryza sativa*	Busset *et al*. (unpublished)	
*P. caribensis*	ABIP 638	ABIP 638	N18-NS	*Oryza sativa*	Busset *et al*. (unpublished)	
*P. fungorum*	LMG 16226	ABIP 114		White-rot fungus *Phanerochaete chrysosporium*		
*P. fungorum*	LMG 18809	ABIP 115		Unclear, probably clinical isolate		
*P. fungorum*	LMG 19511	ABIP 116		Loamy soil		
*P. fungorum*	LMG 16225	ABIP 21		White-rot fungus *Phanerochaete chrysosporium*		GCA_902833645
*P. graminis*	C4D1M	ABIP 5	LMG 18924	Maize senescent root system		641 736 151
*P. hospita*	LMG 20598			*Oryza sativa*		
*P. kururiensis*	M130	ABIP 1108		*Oryza sativa*		GCF_000341045.1
*P. kururiensis*	KP23	ABIP 117	LMG 19447, JCM 10599	Aquifer sample collected at a trichloroethylene-polluted site		GCF_003986935.1
*P. kururiensis*	ABIP 2636	ABIP 2636		*Oryza sativa*	[111]	
*P. kururiensis-like*	HAMBI_2494	ABIP 2425		Human patient		GCF_034424375.1
*P. nodosa*	LMG 23741			*Mimosa scabrella*, root nodules		
*P. phymatum*	STM815	ABIP 1		*Machaerium lunatum* root nodules		GCA_902833665
*P. phytofirmans*	PsJN	ABIP 23		Surface-sterilized onion roots		GCA_000020125
*P. sabiae*	ABIP 445	ABIP 445	P22-NS	*Oryza sativa*	Busset *et al*. (unpublished)	
*P. sabiae*	ABIP 630	ABIP 630	N64-ST	*Oryza sativa*	[110]	
*P. sabiae*	BR3407	ABIP 14	LMG 24235	*Mimosa caesalpinifolia*		GCA_030412785
*P. sacchari*	ABIP 2689	ABIP 2689	MSBF4IS872Fbend	*Oryza sativa*	Busset *et al*. (unpublished)	
*P. sacchari*	LMG 19450	ABIP 118		Sugarcane roots		GCA_000785435
*P. terricola*	LMG 20594			Agricultural soil at 30–60 cm depth		GCA_902833815
*P. tropica*	LMG 22274	ABIP 120		Sugarcane roots		GCA_900109265
*P. tropica*	ABIP 2222	ABIP 2222	P1-3 PCAT	*Oryza sativa* Zonghua	Busset *et al*. (unpublished)	
*P. tropica*	ABIP 659	ABIP 659	37 991, PSP659	*Oryza sativa*	[110]	GCF_914484835.1
*P. tuberum*	LMG 21444			*Aspalathus carnosa*, root nodule		8 111 932 081
*P. unamae*	MTI-641	ABIP 119	DSM 17197, ATCC BAA 744, CIP 107921, LMG 22722	*Zea mays* cv. Landrace, rhizosphere		2 510 917 013
*P. unamae*	ABIP 457		N4-NS	*Oryza sativa*	Busset *et al*. (unpublished)	

a Where a genome sequence has been released for a strain, the designation used in this study is the one under which the sequence has been released.

b Accessions given are for the strains analysed bioinformatically in this study and are for the Joint Genome Institute (JGI) or National Center for Biotechnology Information (NCBI) databases.

### Swimming motility and growth at 37°C are prevalent throughout the *Burkholderia s.l.*

Of the 76 strains tested, 72 exhibited swimming motility, and 53 were able to traverse the entire plate within 3 days. Whereas all members of the *Burkholderia s.s.* showed very high levels of motility, the other *Burkholderia s.l.* genera showed significantly greater variability (Brown–Forsythe test, *P* = 7 × 10^−7^), with a significant trend towards reduced motility (Mann–Whitney test, *P* = 3 × 10^−7^,Table 2). Growth at 37°C is a prerequisite for establishing infections in mammals, and of the 76 strains in our panel, 70 grew at this temperature, including all *Burkholderia s.s.* strains.

**Table 2:** Pathogenicity and phenotypic characteristics of the strain panel.

**Strain** ^**a**^	**% Galleria dead at 72 h** ^**a**^	**37°C** ^**b**^	**Swimming diameter (mm)** ^**c**^	**CAS halo (mm)** ^**c**^	**Protease halo (mm)** ^**c**^	**Antifungal ZOI (mm)** ^**c**^	**Antibacterial halo (mm)** ^**c**^	**Oxalate degradation** ^**b**^
*P. tuberum* LMG21444	17	0	0	0	0	0	0	1
*P. bryophila* 1S18	33	0	85	3	0	8	4	1
*P. megapolitana* A10	17	0	0	1	0	5	0	1
*P. megapolitana* LMG23650	7	0	0	1	0	0	0	1
*P. phytofirmans* PsJN	27	0	85	4	0	0	0	1
*P. terricola* LMG20594	40	1	77	1	0	0	0	1
*P. fungorum* LMG16226	63	1	85	3	0	0	0	1
*P. fungorum* LMG18809	23	0	85	3	0	0	0	1
*P. fungorum* LMG19511	63	1	85	2	0	0	0	1
*P. fungorum* LMG16225	93	1	85	2	0	0	0	1
*P. graminis* C4D1M	37	1	85	2	0	0	0	1
*P. phenazinium* S18	43	1	0	7	0	1	0	0
*P. caribensis* LMG18531	70	1	85	1	0	0	0	1
*P. caribensis* ABIP1334	23	1	85	1	0	0	0	1
*P. caribensis* ABIP462	73	1	85	1	0	0	0	1
*P. caribensis* ABIP477	60	0	85	0	0	0	0	1
*P. caribensis* ABIP478	93	1	85	1	0	0	0	1
*P. caribensis* ABIP638	40	1	76	1	0	0	0	1
*P. hospita* LMG20598	20	0	28	0	0	0	0	1
*P. sabiae* BR3407	0	0	85	1	0	0	0	1
*P. sabiae* ABIP445	23	0	85	2	0	0	0	1
*P. sabiae* ABIP630	43	0	85	2	0	0	0	1
*P. phymatum* STM815	20	1	74	1	0	0	0	1
*P. tropica* LMG22274	100	1	52	1	0	0	0	1
*P. tropica* ABIP659	13	0	60	1	0	0	0	1
*P. tropica* ABIP2222	100	1	42	2	0	0	0	1
*P. nodosa* LMG23741	0	0	25	1	0	0	0	1
*P. sacchari* LMG19450	47	1	68	0	0	0	0	1
*P. sacchari* ABIP2689	10	1	85	0	0	0	0	1
*P. unamae* MTI-641	50	1	43	1	0	0	0	1
*P. unamae* ABIP457	40	1	29	1	0	0	0	1
*P. kururiensis* M130	7	1	55	1	0	0	0	1
*P. kururiensis* KP23	20	1	73	2	0	0	0	1
*P. kururiensis* ABIP2636	100	1	78	0	0	0	0	1
*P. kururiensis*-like HAMBI_2494	33	1	29	1	0	0	0	1
*C. cordobensis* LMG27620	7	1	85	1	0	0	0	1
*C. zhejiangensis* OP-1	7	1	50	2	0	0	0	1
*C. insecticola* RPE64	30	1	77	2	0	0	0	1
*C. grimmiae* LMG27580	3	1	85	2	0	0	0	1
*C. sordidicola* LMG22029	17	0	0	1	0	0	0	1
*B. cepacia* ATCC25416	100	1	85	10	0	7	8	0
*B. cepacia* ABIP2356	100	1	85	6	2	11	12	0
*B. cepacia* ABIP441	100	1	85	10	1	7	13	0
Bcc *cepacia*-like ABIP2227	100	1	85	9	2	10	0	0
Bcc *cepacia*-like ABIP2230	100	1	85	5	2	13	11	0
Bcc *cepacia*-like ABIP2232	100	1	85	7	2	8	0	0
Bcc *cepacia*-like ABIP438	100	1	85	7	2	7	0	0
Bcc *cepacia*-like ABIP449	100	1	85	9	2	9	0	0
*B. orbicola* ABIP443	100	1	85	3	2	8	0	0
*B. orbicola* ABIP444	100	1	85	7	1	11	0	0
*B. cenocepacia* H111	97	1	85	8	2	3	0	0
Bcc *latens*-like ABIP2472	100	1	85	6	1	0	0	0
*B. diffusa* ABIP447	100	1	85	5	2	0	0	0
*B. vietnamiensis* ABIP1335	100	1	85	5	0	8	0	0
*B. vietnamiensis* LMG10929	100	1	85	5	0	7	0	0
*B. vietnamiensis* ABIP434	100	1	82	3	0	11	0	0
*B. vietnamiensis* ABIP2229	100	1	85	3	0	6	0	0
*B. vietnamiensis* ABIP436	27	1	85	5	0	7	0	0
*B. vietnamiensis* ABIP440	87	1	85	5	0	10	0	0
*B. vietnamiensis* ABIP670	100	1	85	2	0	10	0	0
*B. vietnamiensis* ABIP2221	100	1	85	6	0	8	0	0
*B. vietnamiensis* ABIP2300	100	1	85	5	0	8	0	0
Bcc *multivorans*-like ABIP2354	100	1	85	6	0	0	0	0
Bcc *multivorans*-like ABIP2226	7	1	85	0	0	0	0	0
Bcc *multivorans*-like ABIP2352	30	1	85	0	1	0	0	0
*Bcc multivorans-like* ABIP2225	57	1	85	0	0	0	0	0
Bcc *multivorans*-like ABIP2353	77	1	85	1	2	0	4	0
Bcc *multivorans*-like ABIP2355	33	1	85	0	0	0	0	0
*B. thailandensis* E264	100	1	85	1	4	4	21	0
*B. plantarii* ATCC43733	97	1	85	5	1	5	0	0
*B. glumae* LMG2196	100	1	85	3	0	0	0	0
*B. glumae* LMG10906	90	1	85	2	0	0	0	0
*B. glumae* NCPPB3923	97	1	85	4	0	0	0	0
*B. gladioli* ATCC10248	100	1	85	2	0	4	0	0
*T. caryophylli* LMG2155	23	1	49	4	0	3	0	1
*R. andropogonis* ICMP2807	20	0	52	0	0	0	0	0

a Value given is the mean of three biological replicates, n = 10.

b Growth was rated as 0 or 1, where 0 represents no growth and 1 represents growth. Three independent biological replicates were tested and all gave the same result.

c Value given is the mean of three biological replicates. For the antifungal assay n = 3, for all others n = 1.

### Antimicrobial activity is largely restricted to the *Burkholderia s.s.*

*Burkholderia s.l.* strains are well known for their antimicrobial activity (see Supplementary Information). To gain further insight into the distribution of antimicrobial activity, we tested the entire strain panel for activity against the plant pathogens *P. carotovorum* and *Fusarium solani*. Antifungal activity occurred significantly more frequently in the *B. s.s.* than in the other genera (Fisher’s exact test, *P* < .0001); 27 strains in total showed antifungal activity, of which 23 belonged to the *Burkholderia s.s.* Outside of the *Burkholderia s.s.*, *T. caryophylli* LMG 2155*, P. bryophila* 1S18, *P. phenazinium* S18, and *P. megapolitana* A10 showed antifungal activity. Antibacterial activity was also detected more frequently in the *B. s.s.*, although the significance was marginal (Fisher’s exact test, *P* = 0.0492). Activity against *P. carotovorum* was observed in only seven strains: six from the *Burkholderia s.s.* and one from the *Paraburkholderia* (*P. bryophila* 1S18) (Table 2)*.* Our results support the previous notion that the production of antimicrobial compounds is mainly restricted to the *Burkholderia s.s.* and that outside this clade, only strains of *P. bryophila, P. megapolitana, P. phenazinium,* and *T. caryophylli* produce antimicrobials [11, 16, 58–60]*.*

### Siderophore production is highest in the *Burkholderia s.s.*

Siderophore production has been found essential for virulence of Bcc members in all animal infection models tested to date [61, 62]. The majority of our panel strains produced siderophores to some extent, as assessed on CAS plates (Table 2). However, siderophore production was significantly more variable across the *Paraburkholderia* strains tested (Brown–Forsythe test, *P* = 2 × 10^−4^). In general, significantly less iron scavenging was observed in the *Paraburkholderia* than in the *Burkholderia s.s.* (Mann–Whitney test, *P* = 5 × 10^−6^)*.* Only four strains of the *Burkholderia s.s.* did not show siderophore production in our assay, all of which had been classified as *Burkholderia multivorans*–like (ABIP 2226, ABIP 2352, ABIP 2225, and ABIP 2355).

In conclusion, our data not only are in line with previous findings that siderophore production contributes to virulence but also indicate that siderophore production alone cannot distinguish pathogenic from nonpathogenic *Burkholderia s.l.*, as several *Paraburkholderia* strains also show activity [61]. Ornibactin production appears to be restricted to the pathogenic clade, whereas diazenium diolate siderophores appear to be primarily produced by plant-associated *Burkholderia s.l.* species.

### Production of metalloproteases is limited to the *Burkholderia s.s.* lineage

Several members of the Bcc produce the broad-spectrum zinc metalloproteases ZmpA and ZmpB, both of which were shown to contribute to the virulence of *B. cenocepacia* in a rat infection model [63, 64]. In our strain panel, proteolytic activity was found to be entirely confined to the *Burkholderia s.s.,* where it was observed in 16 of the 34 strains tested (Table 2), suggesting that proteolytic activity is a strong indicator of strains with elevated pathogenic potential within this lineage. None of the strains from other *Burkholderia s.l.* genera exhibited proteolytic activity.

### Oxalate utilization is a conserved characteristic of the plant beneficial and environmental *Burkholderia s.l.*

Oxalate degradation in the *Burkholderia s.l.* has been frequently reported among the *Paraburkholderia* but appears to be absent from the *Burkholderia s.s.* [65]. We extended the earlier work by screening our strain panel for oxalate degradation. In accordance with previous work [65], all *Paraburkholderia* and *Caballeronia* members tested were oxalotrophic, with the exception of *P. phenazinium*. Furthermore, none of the *Burkholderia s.s.* members tested exhibited oxalate degradation, suggesting this trait as a robust marker to distinguish strains with lower versus higher pathogenic potential (Table 2) [65].

### Pathogenic potential in the wax moth larva model is not restricted to the *Burkholderia s.s*.

**Figure 1:**
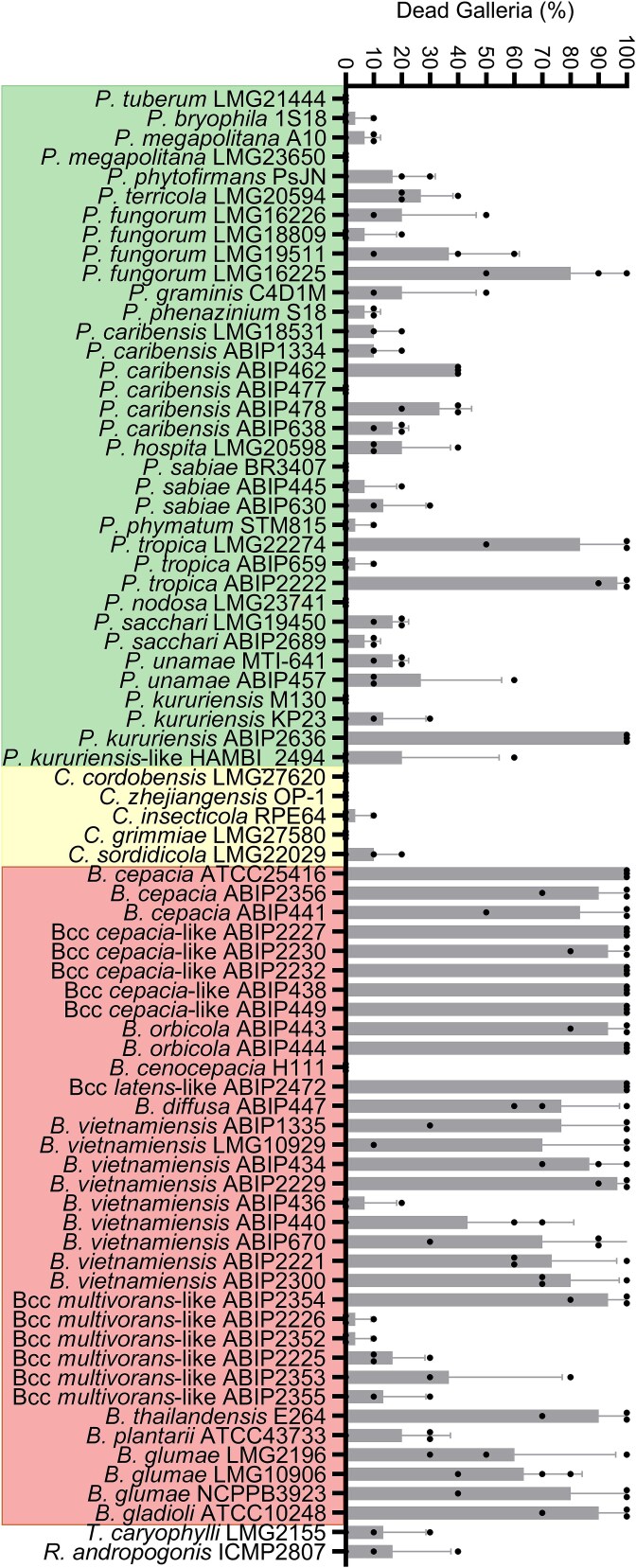
Pathogenicity to wax moth larvae was most consistent within the *Burkholderia s.s.* Percentage of *G. mellonella* larvae that were dead 24 h postinjection with the indicated strain. Bars show the mean of three biological replicates, and error bars represent the standard deviation. Black-filled circles show the result of each individual replicate.

The *G. mellonella* larval model is commonly used to assess strain virulence due to both practical considerations (ease of use, minimal space requirements, and no ethical review required) and immunological relevance (larvae possess an innate immune system) [66]. This model has been extensively applied to study the virulence of *Burkholderia* species [67–70]*.* Testing all panel strains in wax moth larvae revealed that virulence was highly significantly greater among *Burkholderia s.s.* than among *Paraburkholderia* (Mann–Whitney test, *P* = 8 × 10^−8^, at 72 h postinfection)*,* with mean larval mortality of 88% and 41% at 72 and 24 h postinfection, respectively (Table 2, Figs 1 and S2). Whereas the majority of strains exhibiting high pathogenic potential belonged to the *Burkholderia s.s*., several strains from other genera also caused substantial larval mortality, including *P. fungorum* LMG 16225, *P. kururiensis* ABIP 2636, and *P. tropica* strains LMG 22274 and ABIP 2222 (Fig. 1).

As mentioned previously, only four Bcc strains did not show siderophore activity (ABIP 2226, ABIP 2352, ABIP 2225, and ABIP 2355) (Table 2). These four also displayed markedly lower pathogenicity in *G. mellonella* compared to the other Bcc strains (Mann–Whitney test, *P* = 2 × 10^−4^). Attempts to amplify the origin of replication of the pC3 megaplasmid in these strains, which is known to be important for virulence and siderophore production [71], were unsuccessful, suggesting that this replicon had been lost from these four strains. Bioinformatic analysis confirmed that the two sequenced strains (ABIP 2225 and ABIP 2226) indeed lack the pC3 megaplasmid, providing a rationale for both the absence of siderophore activity and reduced pathogenic potential.

### Principal component analysis separates *Burkholderia s.s.* from the other genera

**Figure 2:**
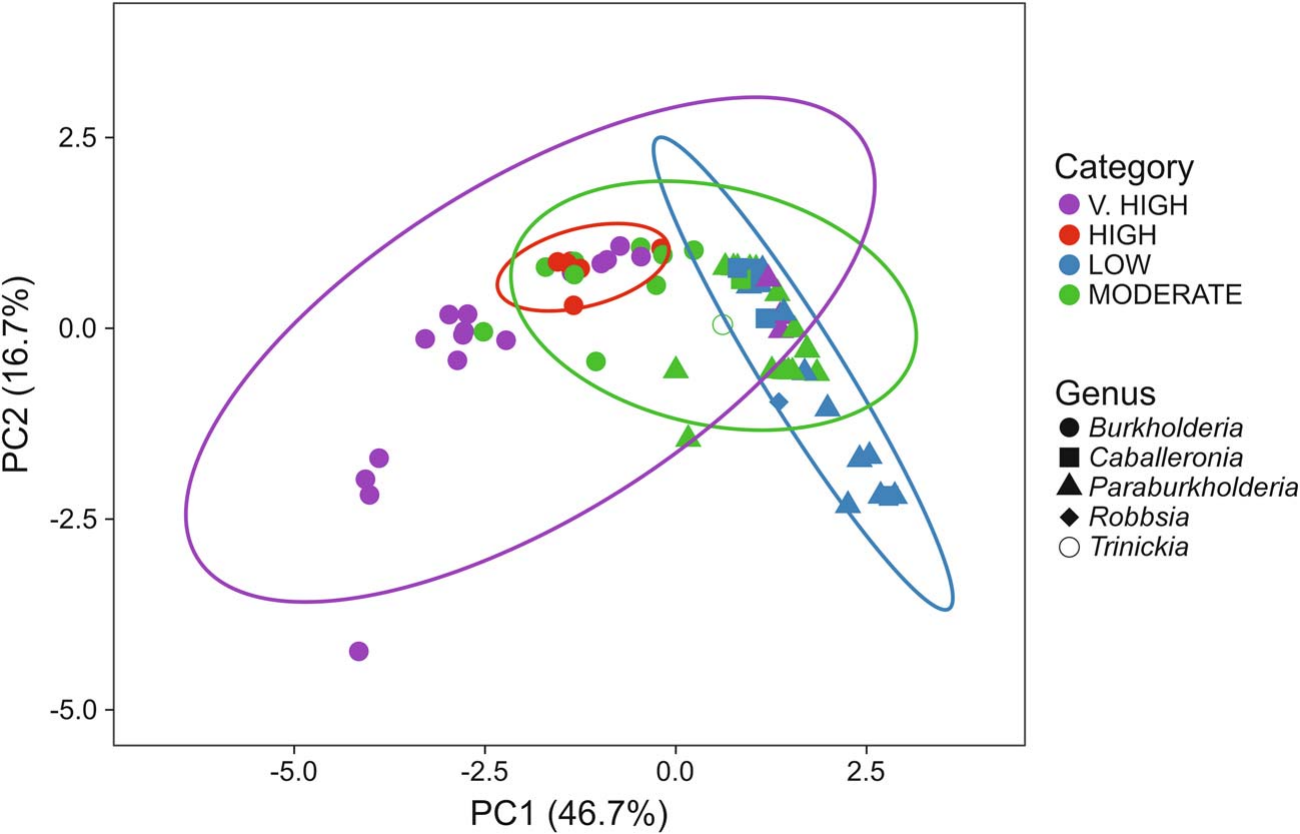
Principal Component Analysis of phenotypic data. The % larvae dead (%D) at 24 and 72 h was classified by quartile. V. high, %D (24 h) ≥3rd quartile; high, %D (72 h) ≥3rd quartile; moderate, %D (72 h) ≥ 1st quartile < 3rd quartile; low, %D (72 h) < 1st quartile. PCA loadings have been provided in the extended data. Analysis was carried out using ClustVis [117].

Principal component analysis of the full strain panel was carried out based on observed phenotypes, with *G. mellonella* infection severity categorized into four groups (low, moderate, high, or very high), and showed that PC1 and PC2 accounted for 63.4% of the variation (Fig. 2). Although *Burkholderia s.s.* separated from other genera, the four *Paraburkholderia* strains causing severe larval infection (*P. fungorum* LMG 16225, *P. tropica* LMG 22274, *P. tropica* ABIP 2222, and *P. kururiensis* ABIP2636) did not segregate distinctly.

### *In silico* investigation of relatedness and secondary metabolites

**Figure 3:**
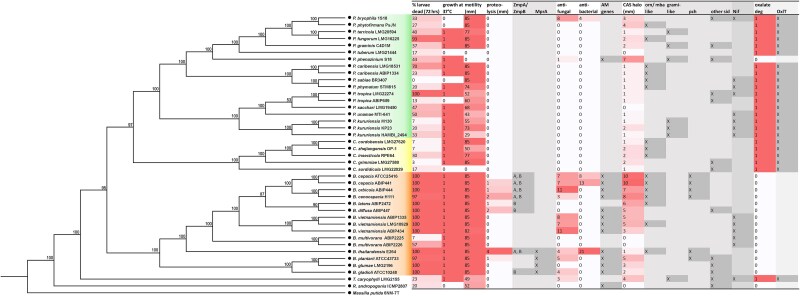
Phylogenetic relationships vs phenotypes and *in silico* analysis across the *Burkholderia s.l.* The cladogram illustrates the strain panel members for which whole genome sequences were available. A cladogram was generated using autoMLST, by concatenated alignment of 100 automatically selected genes present in all species shown. *Massilia putida* 6NM-7T was included as an outgroup. Phenotypic assay results have been shown for the strains depicted, and cells have been shaded depending on the relative strength of the observed result. Assays are as shown in Table 2. The grey-shaded columns indicate the presence of secondary metabolite genes (shaded dark grey when the corresponding gene was present), as found by antiSMASH/BLASTx analysis of the genome sequences, see Materials and Methods for query details. ZmpA/ZmpB analysis: (A) ZmpA encoded; (B) ZmpB encoded. Analysis for siderophore gene clusters was done using MultiGeneBLAST (see Materials and Methods). Orn/mba like, ornibactin/malleobactin/phymabactin-type siderophore cluster; pch, pyochelin; grami-like, gramibactin; other sid, other siderophore (antiSMASH predicted that a siderophore cluster was present, but it did not meet the threshold for designation as one of the specific siderophores).

To link our phenotypic data with genotypic factors, we first constructed a cladogram using autoMLST and combined it with a heatmap of the phenotypic assay results for the 40 sequenced *Burkholderia s.l.* strains (Fig. 3). Secondary metabolic clusters were identified with antiSMASH to pinpoint potential genetic determinants, followed by MultiGeneBLAST analysis for clusters encoding known iron-scavenging and antimicrobial secondary metabolites in *Burkholderia s.l.* [12]. BLASTP was also used to detect clusters associated with the tested phenotypes [12, 17, 29, 71, 72] (Figs 3 and S3).

Proteolytic activity on skimmed milk agar correlated with the presence of *zmpA* and/or *zmpB* homologues. *Burkholderia plantarii* ATCC 43733 exhibited proteolysis despite lacking *zmpA/zmpB*, probably due to it harbouring an MprA protease homologue. Similarly, iron chelation on CAS medium aligned with the presence of siderophore biosynthetic clusters (Fig. 3).

Although many *Burkholderia s.s*. strains produced antimicrobial compounds (Supplementary Discussion), the genes for several remained unidentified [73]. Close homologues of known antifungal gene clusters were identified in all strains exhibiting antifungal activity except *P. bryophila* (Fig. S3)*.* Many encoded compounds, such as phenazines, pyrrolnitrin, gladiofungin A, and gladiolin, have strong antifungal activity and low cytotoxicity [14, 74, 75], and others (bactobolin, glidobactin, and 4-hydroxy-3-methyl-2-alkylquinolines (HMQ)) are being explored as anticancer agents. As fungi are eukaryotes, it is unsurprising that antifungal compound production correlated with increased pathogenicity.

Genomes were also screened for known virulence-related genes using Vfanalyzer [76]. Strikingly, all sequenced genomes, including strains lacking swimming motility, contained multiple flagellar genes (Fig. S4). Extracellular polymeric substance (EPS) and capsule genes, as well as Type VI Secretion System genes, were widespread, whereas other classic virulence factors were mostly restricted to a few strains (Fig. S4), highlighting the relative paucity of typical virulence factors in *Burkholderia s.l.* strains*.*

### Nitrogen fixation within the *Burkholderia s.l.*

Previous research has shown that several *Burkholderia s.l.* species can fix atmospheric nitrogen, either in symbiosis with legumes or under free-living conditions [38]. Although nitrogen fixation is often considered a hallmark of environmental, plant-associated *Burkholderia s.l.* species [39], some *Burkholderia s.s.* strains, most notably *B. vietnamiensis* [77–79], also possess this capability. To assess nitrogen fixation potential in our panel, we screened the sequenced genomes for six core *nif* genes (*nifHDK* and *nifENB*), a robust computational predictor of nitrogen fixation [80]. Thirteen strains carried all six genes, including five Bcc members and seven *Paraburkholderia* strains (Fig. 3). We also screened for the nodulation factor gene, *nodA*, which was detected only in *P. sabiae* BR3407 and *P. phymatum* STM815, both known legume symbionts [81, 82].

### Presence of the *oxlT* antiporter gene is indicative of oxalotrophy

The genes *frc* and *oxc*, encoding formyl-CoA transferase and oxalyl CoA decarboxylase, have traditionally been used to identify putative oxalotrophs by genome mining [65, 83]. However, Robertson and Meyers recently proposed that the oxalate-formate antiporter gene *oxlT* is a more reliable marker, as oxalate degradation can proceed via pathways not involving FRC and OXC, whereas strains lacking *oxlT* cannot degrade oxalate [83]. Our results support this view, as several of the phenotypically oxalotrophic strains in our panel lacked *oxc*, whereas *B. plantarii* ATCC43733, which carries both *frc* and *oxc* but lacks an *oxlT* homologue, did not degrade oxalate (Fig. 3).

### Ornibactin is a *bona fide* virulence factor

Although siderophore production is more common in the *Burkholderia s.s.*, it is not restricted to this lineage (Table 2, Fig. 3). Ornibactin production was largely specific to the pathogenic clade, although *C. insecticola* RPE64 and *Paraburkholderia* sp. 123 harbour plasmid-borne clusters for ornibactin or closely related siderophores [84]. This distribution aligns with previous reports demonstrating that ornibactin is required for virulence in multiple hosts [85] and that, despite the production of several siderophores by some pathogenic strains, ornibactin functions as the primary iron chelator due to its higher iron affinity [86].

**Figure 4:**
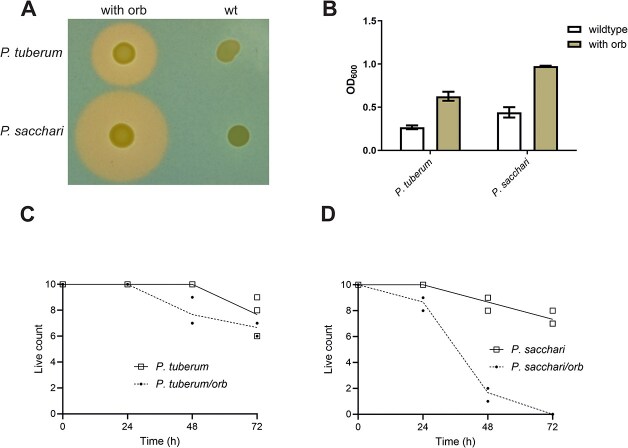
Transgenic expression of the ornibactin cluster conferred siderophore production and virulence upon *P. sacchari.* (A) *P. tuberum, P. sacchari, P. tuberum_Orb,* and *P. sacchari_Orb* exhibiting ornibactin production on CAS agar. (B) Growth of *P. sacchari,* and *P. sacchari*_Orb in M9 minimal medium in the presence of 50 μM bipyridyl. (C, D) *G. mellonella* survival after infection with *P. tuberum, P. tuberum_Orb* (C) and *P. sacchari* and *P. sacchari_Orb* (D). The *Sacchari_Orb* strain had significantly higher virulence than the wildtype *P. sacchari* (Gehen–Breslow–Wilcoxon test *P*-value < 9 × 10^−4^). *P. tuberum_Orb* strain was completely avirulent, indistinguishable from the *P. tuberum* wildtype (*P*-value > .05). Assays were carried out in triplicate (*n* = 10) with similar results.

To assess the contribution of ornibactin to pathogenic potential, we transferred the 64 kb ornibactin biosynthetic cluster from *B. cenocepacia* H111 (using a modified oriTn*7* capture system (Fig. S5) [87]) into the environmental *Paraburkholderia* strains *P. tuberum* LMG 21444 and *P. sacchari* LMG 19450, neither of which produces siderophores on CAS plates or shows high virulence in *G. mellonella* (Figs 3 and 4). Both recombinant strains produced siderophores, and ornibactin production was verified by extraction from culture supernatants (Fig. 4a). Ornibactin production enabled growth of the transgenic strains under iron limitation, whereas the parental strains grew poorly (Fig. 4b), indicating successful heterologous expression. *G. mellonella* infection assays revealed that the recombinant *P. sacchari* strain exhibited markedly increased virulence (Gehen–Breslow–Wilcoxon test, *P* = 9 × 10^−4^), whereas the recombinant *P. tuberum* strain showed low pathogenic potential (Fig. 4c and d). Thus, ornibactin is a *bona fide* virulence factor but requires additional bacterial functions to become effective. Although these functions are present in *P. sacchari,* they are missing from *P. tuberum*. These findings also caution against defining pathogenicity solely by phylogeny, as acquisition of a single genetic locus can substantially increase pathogenic potential in the *G. mellonella* model.

Because iron in soil is largely insoluble, siderophore production is generally thought to enhance fitness [88]. To test this, we compared persistence of the wild-type and ornibactin-producing *Paraburkholderia* strains in soil microcosms over 10 days. No significant differences were observed (paired *t*-test, *P* > 0.05) (Fig. S6). Additionally, a siderophore-deficient *B. cenocepacia* H111 mutant showed similar persistence to the wildtype (paired *t*-test, *P* > 0.05) (Fig. S7), indicating that ornibactin does not contribute to the persistence of these strains in soil. Given that siderophores can adsorb to metal oxides present in many environments [89], we hypothesized that such adsorption reduces their ecological relevance in soil. Adding purified ornibactin and pyochelin to soil microcosms and analysing dissolved versus adsorbed fractions after 10 days revealed that both siderophores predominantly adsorbed to soil particles, with only small amounts remaining soluble (Fig. S8). This supports the idea that siderophore adsorption may limit their utility for iron acquisition in soil environments.

### Expression of the antifungal agent pyrrolnitrin does not increase the pathogenic potential of *P. tuberum* or *P. sacchari*

As few *Paraburkholderia* strains produce antimicrobial compounds, we hypothesized that heterologous expression of pyrrolnitrin could enhance the biocontrol potential of environmental *Paraburkholderia* species. We therefore transferred the *prnABCD* operon of *B. lata*, which encodes pyrrolnitrin biosynthesis from tryptophan [29, 90–92], into *P. tuberum* and *P. sacchari*. Both recombinant strains displayed antifungal activity, whereas the parental strains did not (Fig. S9). Although pyrrolnitrin reportedly both repels and kills nematodes (146), *G. mellonella* infection assays revealed no significant increase in pathogenic potential of the engineered strains (Gehen–Breslow–Wilcoxon test, *P* > 0.05), consistent with the compound’s low cytotoxicity. These findings demonstrate that environmental strains can be engineered to produce antimicrobial metabolites and thereby boost biocontrol potential, without elevating their pathogenic potential.

## Discussion

We applied a multifaceted strategy, combining bioinformatic, phylogenetic, and phenotypic analyses, to characterize over 70 *Burkholderia s.l.* strains isolated from diverse habitats. This study provides a systematic experimental assessment of pathogenic potential across a phylogenetically broad *Burkholderia s.l.* panel, integrating functional phenotyping with genome-informed interpretation (Figs 3 and S3). Several phenotypic traits aligned strongly with genomic traits, and these features correlated with their adaptation to particular ecological niches.

Two previous studies have examined lifestyle-associated genes in the *Burkholderia s.l.* exclusively *in silico* [93, 94]. Jia and Lu used comparative genome analysis to examine a panel of *Burkholderia s.s*. strains, concluding that virulence-related genes, such as those encoding toxoflavin and endopolygalacturonase, were common in plant-pathogenic *Burkholderia* but rarely found in endophytes. In contrast, antimicrobial genes involved in niche competition, particularly occidiofungin and cepacin A, were enriched in endophytic strains [94]*.* Guerrero-Egido and colleagues have developed an *in silico* tool (bacLIFE) to classify lifestyle genes, and mutational analyses validated several predicted functions [93]. In contrast to these predominantly computational approaches, our study is grounded in extensive experimental screening of candidate phenotypic and genetic markers associated with pathogenic potential and biocontrol capacity.

Our results largely support the view that *Paraburkholderia* comprises beneficial environmental species, whereas *Burkholderia s.s.* contains mostly strains with elevated pathogenic potential but with some notable exceptions. Experimentally determined pathogenic potential in the *G. mellonella* model was most consistently associated with *Burkholderia s.s.*, yet four *Paraburkholderia* strains (*P. kururiensis* ABIP 2636, *P. fungorum* LMG 16225, and *P. tropica* LMG 22274 and ABIP 2222) exhibited high pathogenic potential, killing nearly all larvae within 24 h. However, another *P. tropica* strain, ABIP 659, showed much lower virulence (Fig. 1). Comparative genomic analyses revealed that LMG 22274 possesses a gramibactin-like siderophore cluster, whereas ABIP 659 encodes an alcaligin-like siderophore (Fig. 3). Gramibactin shows a higher Fe(III) affinity [95], which probably underpins the observed differences in *G. mellonella* pathogenicity. Conversely, some *B. multivorans*–like strains lacking the pC3 virulence megaplasmid showed markedly reduced pathogenicity (Figs 1 and S2). These findings reinforce the importance of strain-level experimental evaluation rather than taxonomic inference.

Several phenotypes correlated strongly with phylogenetic placement. Proteolytic activity, consistently linked to virulence in prior studies [62–64, 96–98], was exclusively associated with strains exhibiting higher pathogenic potential (Table 2, Fig. 3). This association, together with genomic linkage, identifies protease activity as a practical phenotypic indicator of elevated pathogenic potential. Production of the siderophore ornibactin emerged as another reliable marker of elevated pathogenic potential, and its functional relevance was experimentally demonstrated (Figs 3 and 4). Heterologous expression of the ornibactin cluster substantially increased the pathogenic potential in *G. mellonella* for *P. sacchari* LMG 19450, whereas *P. tuberum* LMG 21444 still showed low pathogenic potential despite producing ornibactin (Fig. 4). This indicates that ornibactin alone is not sufficient for virulence and provides mechanistic support for the use of genetic loci as predictive markers of pathogenic potential.

Siderophores have been reported to play important ecological roles in iron competition and fungal suppression in the rhizosphere [99], but this does not appear to apply to ornibactins. Ornibactins produced by *B. contaminans* MS14 display antibacterial but not antifungal activity, and ornibactin derivatives from *B. catarinensis* likewise lack antifungal effects [99, 100]. Taken together, current evidence indicates that ornibactins primarily contribute to bacterial competition rather than antifungal suppression. Furthermore, our soil microcosm data show that ornibactin and pyochelin are strongly adsorbed to soil particles and dispensable for growth under these conditions (Figs S6–S8), supporting a host- rather than soil-associated role. Our data therefore suggest that ornibactins are particularly important in host environments where iron must be acquired from high-affinity host proteins such as transferrin and lactoferrin [61, 86, 101], consistent with the strong correlation between ornibactin production and experimentally determined pathogenic potential (Fig. 3).

Oxalate degradation was observed in the *Paraburkholderia* and *Caballeronia* clades*,* as well as in the *Trinickia* member tested, but not in the *Burkholderia s.s.* (Table 2, Fig. 3)*,* supporting and broadening its proposed status as a hallmark of the *Paraburkholderia* [65]. Oxalic acid can accumulate in soils as calcium oxalate derived from plant material degradation, where it acts as a strong metal chelator and may reduce mineral availability [83]. However, oxalotrophic bacteria metabolize oxalate, converting calcium oxalate to carbonate and thereby increasing soil pH and improving soil properties [83, 102]. Oxalotrophy contributes to plant mutualism, as demonstrated by impaired root colonization of an *frc* mutant of *P. phytofirmans* PsJN [65]. Conversely, some *Burkholderia s.s.* members produce oxalate via the enzymes ObcA and B or Obc1, stabilizing intracellular pH in the stationary phase [103, 104].

Nitrogen-fixing, legume-nodulating bacteria were originally thought to be restricted to members of the Alphaproteobacteria until two *Paraburkholderia* species isolated from root nodules of papilionoid legumes were shown to carry nodulation genes. Subsequent work identified several nodulating and nonsymbiotic diazotrophic *Paraburkholderia* strains capable of fixing nitrogen in soil- or plant-associated environments [38]. Some *Burkholderia s.s.* strains, notably *B. vietnamiensis* [77–79], also possess this capability. Screening for core *nif* genes revealed 13 positive strains, including seven *Paraburkholderia* and five Bcc members, indicating that nitrogen fixation alone is not predictive of low pathogenic risk, as most *B. vietnamiensis* strains exhibited substantial pathogenic potential (Table 2, Fig. 3). Nodulation genes were detected only in known symbionts, suggesting that they may be a more informative indicator of plant-associated lifestyles. These observations emphasize that beneficial ecological traits such as nitrogen fixation cannot be interpreted in isolation and underscore the value of integrated experimental assessment.

The value of our findings depends on the predictive capacity of the *G. mellonella* model. Although this nonmammalian system has limitations, several studies show strong qualitative agreement with murine models (reviewed in [66, 105]) and it has been extensively used to investigate virulence in various *Burkholderia s.s.* strains. Members of the genus *Paraburkholderia* are generally considered to have low pathogenic potential and have been applied in agricultural settings to improve crop yield [106]. Only a few reports describe *Paraburkholderia* strains isolated from infected patients, including *P. fungorum* and *P. tropica* [107–109]. Intriguingly, strains of these two species were also among the *Paraburkholderia* strains with the highest pathogenic potential in *G. mellonella*, whereas most strains showed low pathogenic potential (Fig. 1). These observations strengthen confidence in the model’s relevance for strain-level risk assessment.

Our results demonstrate that strain-level variation precludes the categorical assignment of *Burkholderia s.l.* isolates as pathogenic or beneficial solely based on taxonomy. Although this uncertainty has been used to justify the general ban of *Burkholderia* strains in agricultural applications, similar ambiguity exists in genera such as *Escherichia coli,* which includes both harmless commensal strains that are part of the normal human microbiota and pathogenic strains responsible for acute intestinal and extraintestinal infections in humans and many animal hosts [110]. Similarly, *Bacillus, Pseudomonas, Clostridium*, and *Streptococcus* all include both beneficial and pathogenic strains [111–115]. Yet these genera are not excluded from clinical or biotechnological use, underscoring the need for strain-specific evaluation rather than taxonomic generalization.

Finally, our work shows that environmental *Paraburkholderia* strains can be engineered to produce pyrrolnitrin without increasing pathogenic potential (Fig. S9), consistent with previous demonstrations of heterologous production of the antifungal polyynes caryoynencin and cepacin in *P. phytofirmans* [116]. We further show that Bcc strains lacking the pC3 megaplasmid are attenuated, in agreement with earlier work reporting reduced virulence in mice following pC3 removal from a biocontrol-active *B. ambifaria* strain without loss of plant protection [13]. Collectively, our findings identify phenotypic traits and genomic markers that can guide rational selection or engineering of strains combining strong biocontrol capacity with low pathogenic risk.

Altogether, the experimentally validated trait set identified here constitutes a functional blueprint for preliminary strain-level risk assessment and represents a step toward predictive deployment of *Burkholderia s.l.* in biotechnology and agriculture.

## Supplementary Material

Supplementary_material_wrag081

## Data Availability

All data underlying this article are either available in the article and its online supplementary material, or at the GenBank Nucleotide database (https://www.ncbi.nlm.nih.gov/genbank/) or Joint Genome Institute (https://jgi.doe.gov/) and can be accessed using the unique identifiers provided in Table 1 of the manuscript.
