# Supplementary material for: Identifying phenotypic and genetic traits for assessing pathogenic potential and biocontrol capacity in Burkholderia sensu lato strains
Source: ISME J. 2026 Apr 10;20(1):wrag081. doi: 10.1093/ismejo/wrag081 (PMC13184526; doi:10.1093/ismejo/wrag081)
Supplement: Supplementary_material_wrag081 [file supplementary_material_wrag081.zip › SupplementaryInfo.pdf]

Supplementary Information

Identifying phenotypic and genetic traits for assessing pathogenic potential and biocontrol capacity in *Burkholderia sensu lato* strains

Kirsty Agnoli[1*], Anugraha Mathew[1*], Stefano Gualdi[1], Sarah Paszti[1], Lionel Moulin[2], Annette Vergunst[3], Peter Mergaert[4] and Leo Eberl[1,#]

Supplementary Methods

CAS assay for the production of siderophores

The production of siderophores was assessed using the Chrome Azurol (CAS) assay [1], which was modified as some of the strains tested were unable to grow on the original medium. 10 ml iron solution (1 mM $FeCl_3$, 10 mM HCl) was carefully mixed with 50 ml freshly prepared 2 mM chrome azurol S (CAS). The resulting solution was gently mixed with 40 ml 5 mM HDTMA (hexadecyltrimethylammonium bromide) to obtain a CAS/Fe^{3+}/HDTMA complex, which was sterilised by autoclaving. 50 ml modified M9 salt solution (1.5 g KH_2PO_4, 2.5 g NaCl, and 5 g NH_4Cl in 50 ml d.H_2O) was diluted in 375 ml d.H_2O. 15.12 g PIPES (piperazine-N,N'-bis(2-ethanesulphonic acid)) was added slowly to this solution with constant stirring. This was dissolved by adding NaOH to bring the pH to 6.8, and the total volume was adjusted to 435 ml (higher pH than 6.8 produces green medium, rather than the desired blue). 7.5 g Bacto agar was added to give the Modified M9 Medium, which was autoclaved and allowed to cool to approximately 50 °C. 15 ml 10 % (w/v) casamino acids, 5 ml 20 % glucose solution, 1 ml 1M $MgSO_4$ and 50µl 1M $CaCl_2$ were then added to the Modified M9 Medium and mixed to combine, before gently adding 50 ml CAS/Fe^{3+}/HDTMA complex along the side of the bottle with sufficient agitation to mix thoroughly. This medium was poured into Petri dishes. After drying, 20 µl spots of overnight culture were inoculated onto the plates and incubated at 30 °C for 24 hours. Siderophore production was observed as a halo around the spotted colony where the siderophores had removed the complexed iron.

Transfer of the 35 kb ornibactin biosynthetic gene cluster

We used the oriTn7 capture system developed for *B. pseudomallei* [2], with slight modifications, to specifically transfer the entire ornibactin locus of around 35 kb into *Paraburkholderia* strains (Fig. S5). Briefly, the ornibactin (*orb*) gene cluster was flanked by parallel copies of an origin of transfer (*oriT*) from plasmid RK2, along with a plasmid replication origin. Providing transfer genes *in trans* enabled the specific transfer of the *oriT*-flanked region to *Escherichia coli*. During this process, site-specific recombination at the *oriT* sites generated a plasmid carrying the *orb* cluster, which stably

replicated from the inserted origin of replication (in this case, the F origin carried on a BAC cloning vector) [3]. All strains, primers and plasmids used are detailed in the Supplementary Material (Tables S1-S3). The detailed procedure was as follows.

1. oriTn7 capture of flanking regions of the ornibactin locus. Initially, left and right flanking regions of the ornibactin biosynthetic locus of *B. cenocepacia* H111 [4] were amplified by PCR and cloned into the pDONR221 gateway donor vector. The pDONR221 vector carrying flanking regions was then recombined with the right and left capture vectors [2]. The pUCTCAPR-GW right capture vector was modified to replace the zeocin resistance marker with a trimethoprim resistance cassette to counter-select against *B. cenocepacia* H111. LR recombination between the right flanking fragment and pUCTCAPR-GW1 was performed using LR Clonase II (Invitrogen life technologies) according to the manufacturer's instructions. The resulting recombinant clones were selected using gentamicin and further confirmed by trimethoprim resistance as well as restriction analysis using *PstI* and *BamHI*. Since the left capture vector encoded a kanamycin resistance marker, as did the entry vector pDONR221, the left flanking region including the *attL* sites was PCR amplified using M13fwd and M13rev primers and then recombined with the pFTCAPL vector using the LR clonase (Invitrogen life technologies). Lucigen's BAC-optimized Replicator v2.0 *E. coli*, that contain the inducible plasmid replication initiator gene *trfA,* was used as the cloning host and the medium was supplemented with 0.2% arabinose for induction of replication from the origin of replication of the pFTCAPL vector before plasmid purification.

2. Construction of a merodiploid donor strain. E. coli S17 was transformed with the right and left capture vectors prior to biparental conjugation with *B. cenocepacia* H111. Merodiploid clones were selected using gentamicin and trimethoprim. The right vector captured merodiploid clone was then used as the recipient for a second conjugation to homologously recombine the left capture vector to obtain the double merodiploid clone. This second conjugation resulted in directly oriented *oriT* sites flanking the *orb* locus in the H111 genome. Correct recombination of right and left capture vectors was confirmed by PCR using primer pairs as mentioned in the Table S2.

3. pOrnibactin-Tn7 vector capture mating. *B. cenocepacia* H111 merodiploid strain harboring two *oriT* sites was used as a donor in a triparental mating. The broad host range conjugation helper, pBBRK2013 [2] facilitated the transfer of the *orb* locus into the recipient *E. coli* replicator strain from the first *oriT* site flanking the *orb* locus until the second *oriT* site, thereby generating pOrnibactin-Tn7, a circular mini F replicon-based plasmid containing the *orb* locus. *E. coli* exconjugants were selected on LB plates and checked for trimethoprim, streptomycin, ampicillin and kanamycin resistance. Ex-conjugants were streaked for single colonies on LB containing X-gluc and the blue colonies were further checked for kanamycin resistance as well as gentamicin sensitivity. To confirm the correct construction of pOrnibactin-Tn7, various PCRs using multiple sets of primers along the

ornibactin locus as well as modified gel Eckhardt electrophoresis [5] was performed. For Eckhardt gel electrophoresis, overnight cultures of the *E. coli* replicator strain harbouring the pOrnibactin-Tn7 vector were resuspended in fresh LB medium and grown to an OD_{600} of approximately 0.6. *E. coli* carrying the 52 kb pRK2013 plasmid was used as a positive control. 150 µl of culture was washed with 500 µl 0.3% sarkosyl and resuspended in 20 µl of lysis solution (1X SBE [10 mM NaOH, 1 mM EDTA, 29 mM boric acid, pH 8.0], 1 % sucrose, 1 mg lysozyme and 40 µg RNase A). Samples were run on an Eckhardt gel (1× SBE, 0.9% agarose, 0.5% SDS) in 1x SBE buffer at 100 V for 2.5 h. The gel was then stained for 30 minutes in 0.4 µg of ethidium bromide per ml and destained for 10 min in SBE buffer before imaging.

4. Transposition of pOrnibactin-Tn7 vector into the genomes of target strains. The pOrnibactin-Tn*7* vector was transferred into *E. coli* S17 by electroporation and transformants were selected on LB plates supplemented with kanamycin and trimethoprim. The transformants were also checked for gentamicin sensitivity to rule out the presence of the helper plasmid. In the following transposition step, the pOrnibactin-Tn*7* vector was transferred from *E. coli* S17 by conjugation and integrated into *Paraburkholderia tuberum* and *P. sacchari* using pTNS3 as the transposase helper. Ex-conjugants were selected on PIA plates (DIFCO) supplemented with trimethoprim and checked for kanamycin sensitivity to rule out single crossover events. Confirmation of integration of the Tn*7* transposon at the *attTn7* site located downstream of the *glmS* gene was verified by PCR and sequencing using the primer pairs listed in Table S2. Ornibactin production by *P. tuberum* and *P. sacchari* ex-conjugants was tested by chrome azurol S assay as well as by extracting ornibactin from bacterial cultures, as described previously [6]. For ornibactin extraction, *P. sacchari* orb was grown for 40 hours in IFS medium containing 5mM ornithine. 100 ml culture supernatants were concentrated under vacuum and saturated with NaCl followed by extraction of siderophore-containing residue with a 1:1 ratio of chloroform: phenol. The extract was resolubilised by adding diethyl ether and water (8: 1 ratio) to the mixture. The aqueous phase containing ornibactin was washed three times with ether, allowed to dry and extracted using methanol.

Bioinformatic analyses

The genomes of the 40 sequenced strains were analysed with antiSMASH version 7.0 to gain an overview of their secondary metabolite clusters [7, 8]. Subsequently, the genomes were analysed for their potential to produce specific secondary metabolites known to be produced by the *Burkholderia sensu lato.* BLASTX was used to analyse the genomes for *zmpA, zmpB, mprA, nifHDK and nifENB* [9, 10]. These genes were considered present where the BLASTX match had more than 80% identity over the whole length of the query protein sequence. The core genes of the known *Burkholderia sensu lato* antifungal clusters specifying phenazine [11], glidobactin [12], occidiofungin [13], AFC-BC11 [14], cepacin [15], HMQ [16, 17], pyrrolnitrin [18, 19], fragin [20] and lagriamide [21], as well as

the known siderophore clusters (ornibactin/malleobactin/phymabactin [4, 22], gramibactin [23, 24], pyochelin [4], cepaciachelin [4, 25]), were used as queries for MultiGeneBLAST, using settings as follows; synteny eight 0.5, minimal sequence coverage 30, minimal % ID 50, max distance 50 kb (see extended data for query sequences). These settings were more stringent than the defaults but still detected the related malleobactin- and phymabactin-type siderophores using the ornibactin cluster from *B. cenocepacia* H111 as query. Hits were only counted if at least 70 % of the query cluster (by length) was present, although in some cases the genes were split over multiple loci. In the *in silico* analysis for fragin production, matches were only scored if homologues of *B. cenocepacia* H111 *hamB, hamF* and *hamG* were present, as many strains have a subset of the genes required for fragin biosynthesis, which specify a diazeniumdiolate signal molecule [20, 26].

Cladogram construction was carried out for all 40 genomes using autoMLST [27]. The tree was constructed using 100 automatically selected, concatenated loci present in all the genomes, including the *Massilia putida* 6NM-7T outgroup. Phylogenetic tree construction was carried out using using AutoMLST2 by concatenated alignment of 87 automatically selected loci present in the genomes used [28]. Where available, strain panel sequences were used, with the addition of four genomes (*P. megapolitana* LMG23650, *P. hospita* DSM171164, *P. nodosa* DSM21604 and *B. diffusa* LMG24065) to give one representative for each species included in our panel. *Ralstonia solanacearum* K60 and *Lysobacter* sp. 031216755 were included as outgroups. VFanalyzer [29] was used to search the sequenced genomes for homologues of known virulence related genes (searches carried out on 14-16th January 2024). ClustVis was used for principle component analysis of the phenotypic data for the entire panel of 76 strains [30]. For this, the disease severity in *G. mellonella* was categorised on quartiles of larval mortality percentage in the entire dataset, as follows: very high, ≥3rd quartile at 24h post-infection; high, ≥3rd quartile at 72h post-infection; moderate, between 1st and 3rd quartile at 72h post-infection; and low infection <1st quartile at 72h post-infection. PCA loadings are provided in the Extended Data.

Supplementary Discussion

Antimicrobial compounds produced by members of the *Burkholderia sensu lato*.

Species of the *B. pseudomallei* lineage produce a suite of secondary metabolites, including malleilactone, burkholdacs A-D, bactobolin and thailandamide, many of which are active against prokaryotic and/or eukaryotic cells [31, 32]. In addition, several *Burkholderia sensu stricto* strains have been demonstrated to produce antifungal molecules, which can provide plants with protection against fungal diseases [13-15, 20, 33-35]. Various of these secondary metabolites have been well-studied, although there are many more still to be identified. Known metabolites fall into several classes, namely the non-ribosomal peptides, the polyketides, the hybrid non-ribosomal peptide/

polyketides (NRPS/PKS), the polyynes and the shikimate pathway derivatives [36]. The non-ribosomal peptides burkholdin and occidiofungin of the Bcc are structurally similar molecules with antifungal activity due to their ability to disrupt eukaryotic cell membranes [36]. Two polyketide secondary metabolites are produced by strains of *B. gladioli*; gladiolin and bongkrekic acid. The former is a macrolide antibiotic that inhibits bacterial transcription and also has activity against *Candida albicans*, but shows very little cytotoxicity [37], whereas bongkrekic acid is a highly potent respiratory toxin. Among the NRPS/PKSs are the bacterial protein synthesis inhibitors enacyloxin IIa and bactobolin, with the former being produced by *Burkholderia ambifaria* and *B. gladioli*, whereas the latter was identified in *Burkholderia thailandensis* [38, 39]. Two polyynes are well-known products of the *Burkholderia sensu lato*: cepacin, which is produced by many Bcc members, and caryoynencin, which is produced by *B. gladioli* and also *T. caryophylli* [15, 40]. Polyynes are molecules with a chain of alternating single and triple carbon to carbon bonds terminating in an alkyne group. They are highly unstable and show both antifungal and antibacterial activity by inhibiting acetyl CoA acetyl transferase and disrupting the fungal cell membrane [36, 40]. Another highly potent and broad-spectrum antifungal agent of the *Burkholderia* is the shikimate pathway derivative pyrrolnitrin [18, 41-43]. This is widely distributed through the Bcc and acts as an uncoupling agent in oxidative phosphorylation [42]. In addition, small active molecules such as toxoflavin and tropolone, which are produced by *Burkholderia glumae* and *Burkholderia plantarii*, respectively, show antimicrobial activity [44], and *B. cenocepacia* produces two antifungal metabolites, AFC-BC11 and fragin [14, 20, 26]. Pyrrolnitrin is a broad-spectrum antifungal that inhibits the respiratory chains of various fungi belonging to the *Basiomycota, Deuteromycota* and *Ascomycota* [41, 45]. By contrast, very few strains of the environmental clade have been reported to produce antimicrobial compounds [40, 46-49]. These compounds include phenazines, known to be produced by *Paraburkholderia phenazinium* (and also by some Bcc members), the previously mentioned caryoynencin, produced by *T. caryophilli*, and the NRPS/PKS rhizoxin, a mitosis inhibitor produced by *Mycetohabitans rhizoxinica* [50].

Siderophores produced by members of the *Burkholderia sensu lato*.

Although Bcc strains can produce a range of siderophores, ornibactin is considered the primary siderophore due to its ability to form 1:1 complexes with iron [4, 51-53]. This is further supported by the siderophore profiles of clinical Bcc isolates, in which ornibactin is the most commonly produced siderophore [54]. The biosynthetic gene cluster for ornibactin is highly homologous to that of the structurally similar malleobactin, which is the major siderophore of the pseudomallei group. Whereas the three ornibactin congeners can all form 1:1 complexes with Fe(III), only one of the eight malleobactin congeners, malleobactin E, can form a 1:1 complex, and the remaining seven do not show strong enough iron binding to constitute true siderophores [52]. Malleobactin E exhibits

weaker iron binding than ornibactin, and malleobactin has been found to be dispensable to *B. pseudomallei* for lethal murine melioidosis, as has its other siderophore, pyochelin [55]. Indeed, it appears that *B. pseudomallei* might modulate host iron homeostasis to increase iron availability, bypassing the need for siderophores during pathogenesis [56]. A third member of this siderophore group has been predicted by genome mining. This ornibactin/malleobactin-like siderophore has been termed 'phymabactin' and was found in the *Paraburkholderia phymatum* STM815 genome [22].

Another high affinity siderophore produced by members of the genus *Paraburkholderia* is gramibactin, which carries an unusual diazeniumdiolate moiety and forms a 1:1 complex with Fe(III) [23]. This siderophore, which was originally isolated from *Paraburkholderia graminis* [24], is not classed as a virulence factor, given that the strains that produce it are not of clinical importance. By contrast, given that gramibactin can also liberate nitric oxide (NO), which is important plant hormone regulating various functions in plants, including growth, defence mechanisms, and the formation of symbioses, this siderophore has been suggested to increase plant fitness, root growth, and tolerance towards stress [57]. This study also identified two new types of diazeniumdiolate siderophores, megapolibactins and plantaribactin, in the moss-associated species *Paraburkholderia megapolitana* and the plant pathogen *B. plantarii*. Moreover, a linearized form of gramibactin, trinickiabactin, was recently shown to be produced by *T. caryophylli* [57].

Production of metalloproteases contribute to virulence of members of the *Burkholderia s.s.*

It has been suggested that proteases are particularly important for the infection of mammalian hosts by the *Burkholderia s.s.*, as they were shown to modulate the host immune response by degrading specific tissue components, including collagen and fibronectin, and for obstructing immune proteins, such as neutrophil alpha-1 proteinase inhibitor, gamma interferon, and immunoglobulins [58]. Given that neither non-mammalian hosts nor plants possess advanced immune systems, they should be unaffected by the activities of ZmpA and ZmpB [59]. The transcription of *zmpA and zmpB* is regulated by the LysR-type regulator ShvR and by quorum sensing, leading to differences in expression of the two metalloproteases in different strains, and hence in the relative importance of each protease to a given strain [60, 61]. In addition to ZmpA and ZmpB, *B. pseudomallei* is known to produce the serine metalloprotease MprA, although deletion of *mprA* showed no effect on virulence in nasal, subcutaneous or intraperitoneal murine models of infection [62, 63].

Table S1: Bacterial strains used in this study

Strain name	Genotype/characteristic	Reference
***Escherichia coli* strains**		
Top 10		Invitrogen
CC118 λpir	*Δ*(*ara, leu*)7697 *araD*139 *ΔlacX*74 *galE galK pho*A20 thi-1 *rps*E *rpoB(*RfR) *argE*(am) *recA1* λpir+	[64]
S17	thi *recA* pro hsdR- hsdM+ RP4-2-Tc::Mu-Km::Tn7	[65]
Replicator v2.0	F⁻ *mcrA* Δ(mrr-hsdRMS-mcrBC) endA1 *recA*1 ϕ80d*lacZ*ΔM15 *ΔlacX*74 *araD*139 *Δ*(ara,leu)7697 *galU galK rpsL* (StrR) *nupG* (attL araC-PBAD-trfA250 bla attR) λ–	Lucigen
***Burkholderia sensu lato* strains**		
Burkholderia cenocepacia H111	Clinical isolate, Wild type	[66]
Burkholderia lata 383	Forest soil isolate, type strain	[67]
Paraburkholderia tuberum	Root nodule isolate from tropical legume	[68]
Paraburkholderia sacchari	Soil isolate from sugarcane plantation	[69]
P. tuberum orb	*P. tuberum* carrying ornibactin locus	This study
P. *sacchari* orb	*P. sacchari* carrying ornibactin locus	This study

Table S2: Oligonucleotides used in this study

Primer name	Primer sequence
**Primers used for cloning ornibactin locus into *Burkholderia sensu lato* strains**	
orb_L_attB1_fwd	GGGGACAAGTTTGTACAAAAAAGCAGGCTCTGAGTCAGGCCGTCGATAC
orb_L_attB2_rev	GGGGACCACTTTGTACAAGAAAGCTGGGTAGATCGGGAACGACAGGAT
orb_R_attB1_fwd	GGGGACAAGTTTGTACAAAAAAGCAGGCTCATCAAGGAGGTCGTGCAT
orb_R_attB2_rev	GGGGACCACTTTGTACAAGAAAGCTGGGTCCTTCAATGCTTCCTGCAAT
att_fwd	CCATCAAACCACGTCAAA
Kan_Dn_rev	CGAAATGACCGACCAAGCGA
att_rev	TACGATACACTTCCGCTCA
Kan_Up_fwd	ACGTGTTCCGCTTCCTTTAGC
CAPL_orbL_fwd	ACGACATCAGTTTGCTCCT
**Primers used to exchange the resistance cassette in CAPR-plasmid for ornibactin introduction**	
trp_frt_fwd	GGGGTCTAGAGAATAGGAACTTCGGAATAGGAACTTCCAGTTGACATAAGCCTGTTCGG
trp_frt_rev	GGGGTCTAGAAAGTATAGGAACTTCTTAGGCCACACGTTCAAGTGC
**Primers used to test the insertion of the ornibactin locus into *Burkholderia sensu lato* strains**	
orbS_rev	TGATCGGAAATCGCTGGG
orbG_fwd	ATGACCCTGCTTTCGTTG
orbG_rev	TTCCTGCATGTCGTCGTC
orbF_fwd	ACCTGCTCGATACCTGTC
orbF_rev	GATGAGATCCGGCTTGAC
orbI_fwd	CACATGACGAGTTTCCCG
orbI_rev	GACGCGAAATGCAGCAGA
pvdA_fwd	CGTATTTCGAGGCGTTCT
pvdA_rev	GACGACGAATGGATCACG
orbA_fwd	CGCGTCCTATGTCTACCA
orbA_rev	GGATCGAACAGAACAGCG
orbJ_fwd	GGATCCCGGATGACGAAGGTGCAA
orbJ_rev	CCCGGGGAGGCGGTAGGTATCGAG
**Primers used to check the correct insertion of ornibactin locus upstream of *glmS* gene in *Burkholderia sensu lato* strains**	
cag_fwd (for *B. sacchari*)	GTCATACTGGCCTCCTGATGTCGTC
glmS_fwd (for *B. tuberum*)	CAGCGCAACCGACAGAAA
TN7_L	ATTAGCTTACGACGCTACACCC
**Primers used for the amplification of the Bcc pC3 origin of replication**	
BccRepF	TTCTCCGACCAGATTCGCAG
BccRepR	GCCTTCTTCTTCGGCGACA
**Primers annealed with dsJ23109Rev2 to form a double stranded insert. Bases that form the cohesive ends of the fragment have been shown in bold.**	
dsJ23109For2	**C**agctagctcagtcctagggactgtgctagc**a**
dsJ23109Rev2	**Agctt**gctagcacagtccctaggactgagctagct**ggtac**
**Used to amplify and clone the pyrrolnitrin cluster. Bases homologous to the *B. lata* 383 genome have been shown in uppercase**	
prnBamFor	gcgcggatccTGGGAACCGTGTCATGAGCA
prnXbaRev	gcgctctagaACTAGGACCTTCGACTCACG

Table S3: Plasmids used in this study

Plasmid	Genotype/ description	Reference
pDONR221	Gateway adapted donor vector; attP1 and attP2, ccdB, pUC origin, Cm^R	Invitrogen
pAUC40	Gateway compatible suicide vector: R6K origin, $Strep^R$ and Cm^R, sacB, ccdB, attR1 and attR2	[70]
pRK600	RK2-mob^+ RK2-tra^+, ori ColE1	[71]
pRK2013	RK2 derivative, mob^+ tra^+ ori ColE1	[72]
pSU11	Promoter probe vector, Gm^R	[73]
pFTCAPL-GW	ori*Tn*7 left capture vector; Gateway destination vector, mini-F copy control replicon, Km^R Cm^R	[2]
pUCTCAPR2-Tp^R	ori*Tn*7 right capture vector; Gateway destination vector, pUC replicon, FRT-ZeoR-FRT cassette is swappable by XbaI digest; Tp^R Gm^R Cm^R	This study
pBBRK2013	pBBR conjugation helper plasmid; Gm^R Km^R	[2]
pOrnibactin-Tn7	Mini F replicon carrying the ornibactin locus, Km^R, Tp^R	This study
pDONR-orbL	Directional gateway entry vector carrying left flanking region of ornibactin locus, Km^R	This study
pDONR-orbR	Directional gateway entry vector carrying right flanking region of ornibactin locus, Km^R	This study
pFTCAPL-orbL	LR recombinant of pFTCAPL-GW and left ornibactin flanking fragment, Km^R	This study
pUCTCAPR-orbR	LR recombinant of pUCTCAPR2-Tp^R and right ornibactin flanking fragment, Tp^R, Gm^R	This study
pTNS3	Tn7 transposase helper plasmid, site-specific TnsD-	[74]

	pathway; pir-dependent replicon, Amp^R	
pBBR1MCS-pJ23109	Broad host range plasmid bearing a constitutive moderately active promoter based on BBa_J23109	[75]
pBBR1MCS-pJ23109-prn	pBBR1MCS-pJ23109 with *prn* cluster cloned between BamHI and XbaI sites in the MCS.	This study

Table S4: Characteristics of soil used in microcosm study

Soil characteristics	Value
% organic C	5.71
C:N ratio	14.3
% silt + clay	76
texture class	clay loam
pH	7.18
C min rate	6.244
**Trace metals (mg/l)**	
Al	0.050
Ca	0.200
Fe	0.040
K	0.800
Mg	0.100
Mn	0.020
Na	0.130
Pb	0.050
Zn	0.030

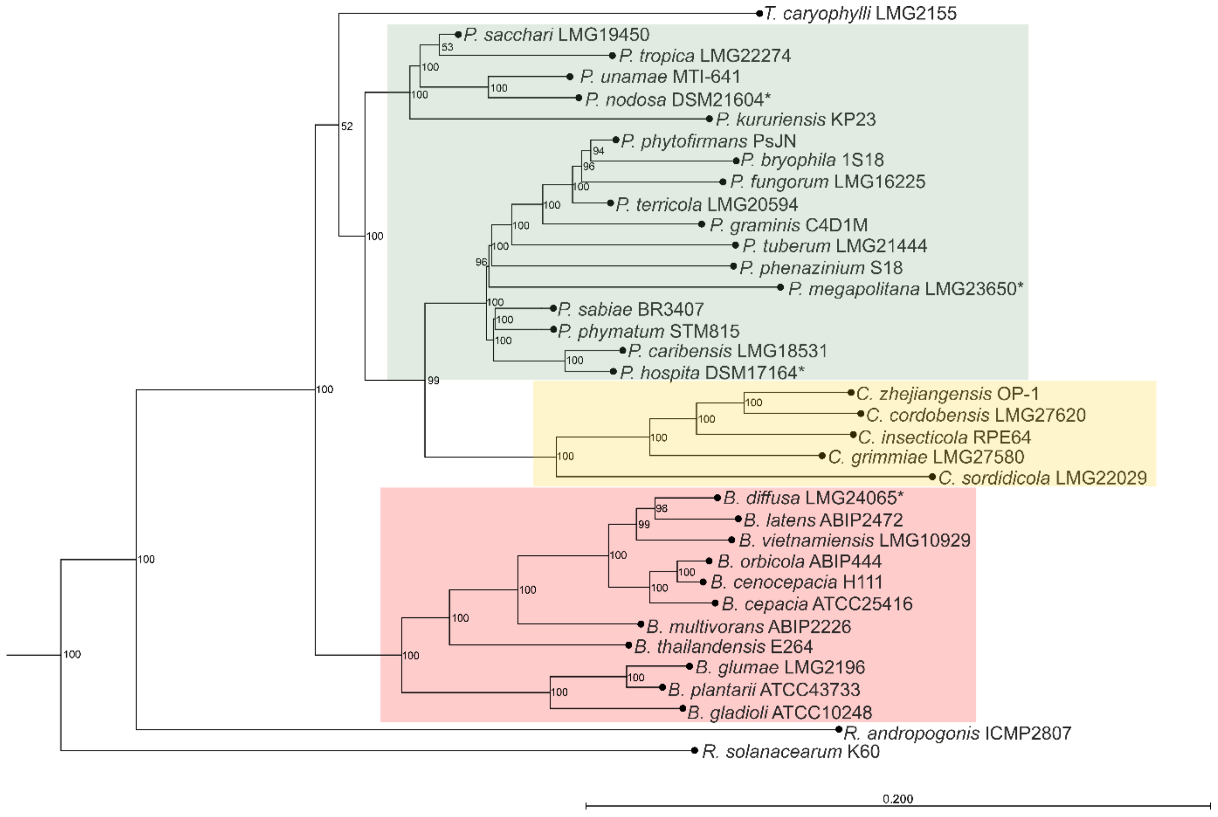

Fig. S1: Phylogenetic tree showing panel species. Tree illustrates one representative for each species included in the strain panel. The *Burkholderis s.s.* has been boxed in red, the *Caballeronia* in yellow, and the *Paraburkholderia* in green. All species shown belong to the *Burkholderia s.l.*. Where available, strain panel sequences were used, with the addition of *P. megapolitana* LMG23650, *P. hospita* DSM171164, *P. nodosa* DSM21604 and *B. diffusa* LMG24065 sequences (indicated on the tree with asterisks). Tree was generated using AutoMLST2 by concatenated alignment of 87 automatically selected loci present in all species shown [28]. *Ralstonia solanacearum* K60 and *Lysobacter* sp. 031216755 were included as outgroups. Tree has been cropped to exclude *Lysobacter* sp. 031216755.

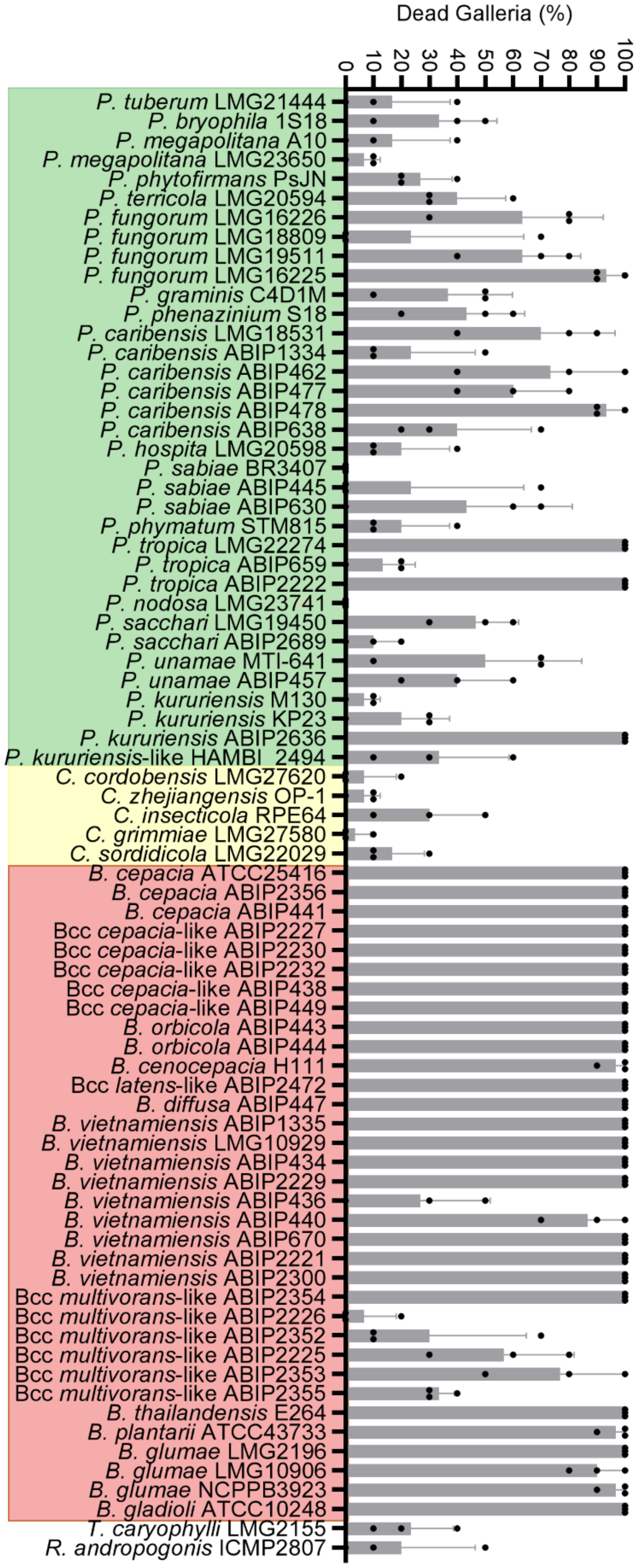

Fig. S2: Pathogenicity to wax moth larvae 72 hours post infection. Percentage of *G. mellonella* larvae that were dead 72 hours post injection with indicated strain. Bars show mean of three biological replicates and error bars represent the standard deviation. Black-filled circles show the result of each individual replicate.

	% larvae dead (72 hrs)	anti-fungal	anti-bacterial	AM genes	phena-zine	AFC	bacto-bolin	cepacin	fragin	gladio-fungin A	gladiolin	glido-bactin	hmq	occidio-fungin	prn
P. phenazinium S18	43	1	0	X	X										
P. sabiae BR3407	0	0	0	X											
C. cordobensis LMG27620	7	0	0	X											
B. cepacia ATCC25416	100	7	8	X		X							X		X
B. cepacia ABIP441	100	7	13	X		X							X		X
B. orbicola ABIP444	100	11	0	X		X			X						X
B. cenocepacia H111	97	3	0	X		X			X						
B. diffusa ABIP447	100	0	0	X				X							
B. vietnamiensis ABIP1335	100	8	0	X				X						X	
B. vietnamiensis LMG10929	100	7	0	X				X						X	
B. vietnamiensis ABIP434	100	11	0	X				X						X	
B. multivorans ABIP2225	57	0	0	X											
B. multivorans-like ABIP2226	7	0	0	X											
B. thailandensis E264	100	4	21	X			X						X		
B. plantarii ATCC43733	97	5	0	X								X			
B. glumae LMG2196	100	0	0	X	X		X								
B. gladioli ATCC10248	100	4	0	X						X	X				
T. caryophylli LMG2155	23	3	0	X											
R. andropogonis ICMP2807	20	0	0			X									

Fig. S3: BLASTN matches for known *Burkholderia* antifungal clusters. Genomes were searched for the following known *Burkholderia* antifungal clusters: (from left to right) phenazine [11], glidobactin [12], occidiofungin [13], AFC-BC11 [14], cepacin [15], HMQ [16, 17], pyrrolnitrin [18, 19], fragin [20], gladiofungin [76], gladiolin [37], bactobolin [39, 77]. The lagriamide and enacyloxin clusters were also included [21, 38], but no matches meeting the criteria for inclusion were found. Strains are only shown if they either showed antifungal activity or if antifungal genes were present.

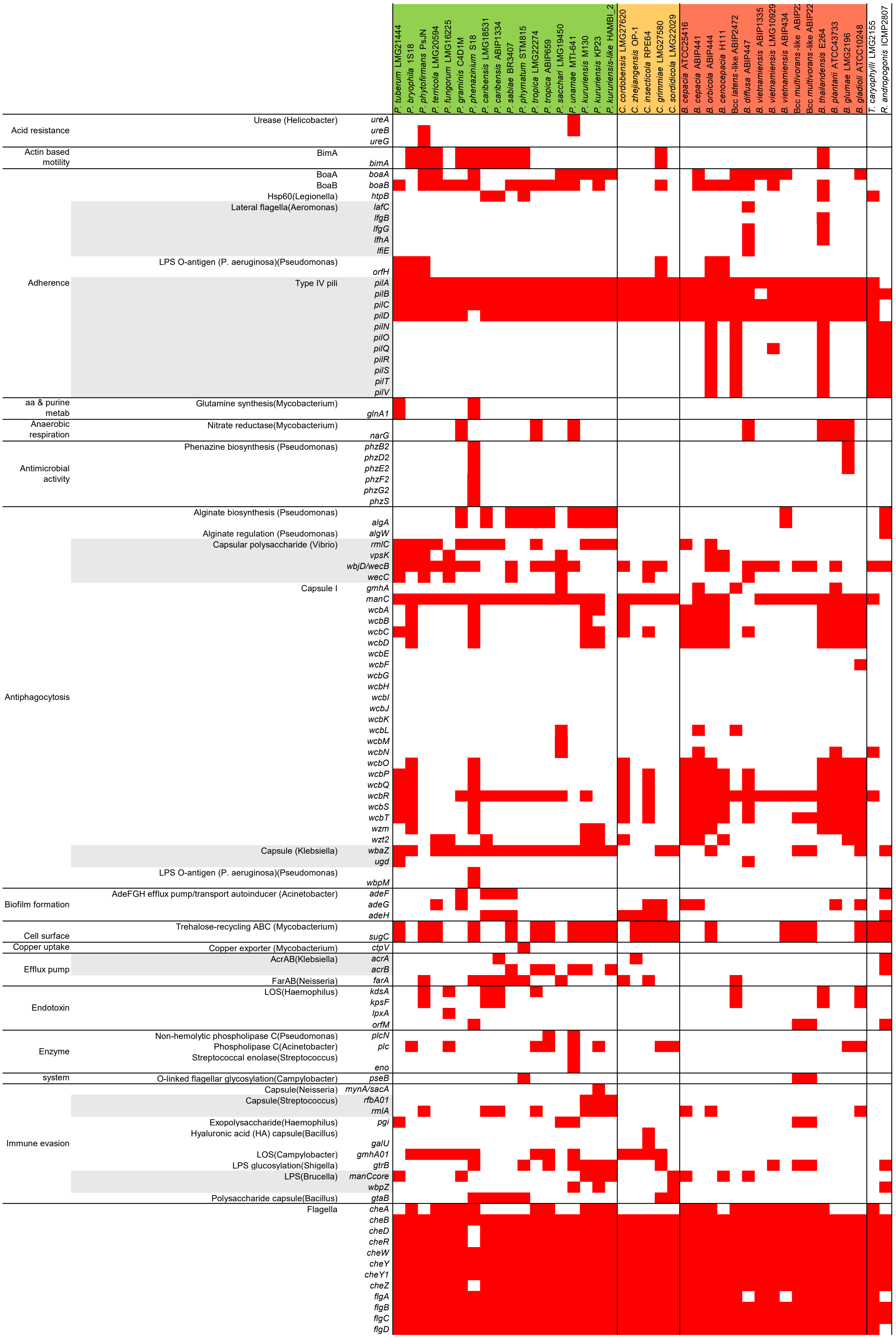

P. tuberum LMG21444
P. bryophila 1S18
P. phytofirmans PsJN
P. terricola LMG20594
P. fungorum LMG16225
P. graminis C4D1M
P. phenazinium S18
P. caribensis LMG18531
P. caribensis ABIP1334
P. sabiae BR3407
P. phymatum STM815
P. tropica LMG22274
P. tropica ABIP659
P. sacchari LMG19450
P. unamae MTI-641
P. kururiensis M130
P. kururiensis KP23
P. kururiensis-like HAMBI_2
C. cordobensis LMG27620
C. zhejiangensis OP-1
C. insecticola RPE64
C. grimmiae LMG27580
C. sordidicola LMG22029
B. cepacia ATCC25416
B. cepacia ABIP441
B. orbicola ABIP444
B. cenocepacia H111
Bcc latens-like ABIP2472
B. diffusa ABIP447
B. vietnamiensis ABIP1335
B. vietnamiensis LMG10929
B. vietnamiensis ABIP434
Bcc multivorans-like ABIP22
Bcc multivorans-like ABIP22
B. thailandensis E264
B. plantarii ATCC43733
B. glumae LMG2196
B. gladioli ATCC10248
T. caryophylli LMG2155
R. andropogonis ICMP2807
Acid resistance
Urease (Helicobacter)
ureA
ureB
ureG
Actin based motility
BimA
bimA
Adherence
BoaA
boaA
BoaB
boaB
Hsp60(Legionella)
htpB
Lateral flagella(Aeromonas)
lafC
lfgB
lfgG
lfhA
lfiE
LPS O-antigen (P. aeruginosa)(Pseudomonas)
orfH
Type IV pili
pilA
pilB
pilC
pilD
pilN
pilO
pilQ
pilR
pilS
pilT
pilV
aa & purine metab
Glutamine synthesis(Mycobacterium)
glnA1
Anaerobic respiration
Nitrate reductase(Mycobacterium)
narG
Antimicrobial activity
Phenazine biosynthesis (Pseudomonas)
phzB2
phzD2
phzE2
phzF2
phzG2
phzS
Antiphagocytosis
Alginate biosynthesis (Pseudomonas)
algA
Alginate regulation (Pseudomonas)
algW
Capsular polysaccharide (Vibrio)
rmlC
vpsK
wbjD/wecB
wecC
Capsule I
gmhA
manC
wcbA
wcbB
wcbC
wcbD
wcbE
wcbF
wcbG
wcbH
wcbI
wcbJ
wcbK
wcbL
wcbM
wcbN
wcbO
wcbP
wcbQ
wcbR
wcbS
wcbT
wzm
wzt2
Capsule (Klebsiella)
wbaZ
ugd
LPS O-antigen (P. aeruginosa)(Pseudomonas)
wbpM
Biofilm formation
AdeFGH efflux pump/transport autoinducer (Acinetobacter)
adeF
adeG
adeH
Cell surface
Trehalose-recycling ABC (Mycobacterium)
sugC
Copper uptake
Copper exporter (Mycobacterium)
ctpV
Efflux pump
AcrAB(Klebsiella)
acrA
acrB
FarAB(Neisseria)
farA
Endotoxin
LOS(Haemophilus)
kdsA
kpsF
lpxA
orfM
Enzyme
Non-hemolytic phospholipase C(Pseudomonas)
plcN
Phospholipase C(Acinetobacter)
plc
Streptococcal enolase(Streptococcus)
eno
system
O-linked flagellar glycosylation(Campylobacter)
pseB
Immune evasion
Capsule(Neisseria)
mynA/sacA
Capsule(Streptococcus)
rfbA01
rmlA
Exopolysaccharide(Haemophilus)
pgi
Hyaluronic acid (HA) capsule(Bacillus)
galU
LOS(Campylobacter)
gmhA01
LPS glucosylation(Shigella)
gtrB
LPS(Brucella)
manCcore
wbpZ
Polysaccharide capsule(Bacillus)
gtaB
Flagella
cheA
cheB
cheD
cheR
cheW
cheY
cheY1
cheZ
flgA
flgB
flgC
flgD

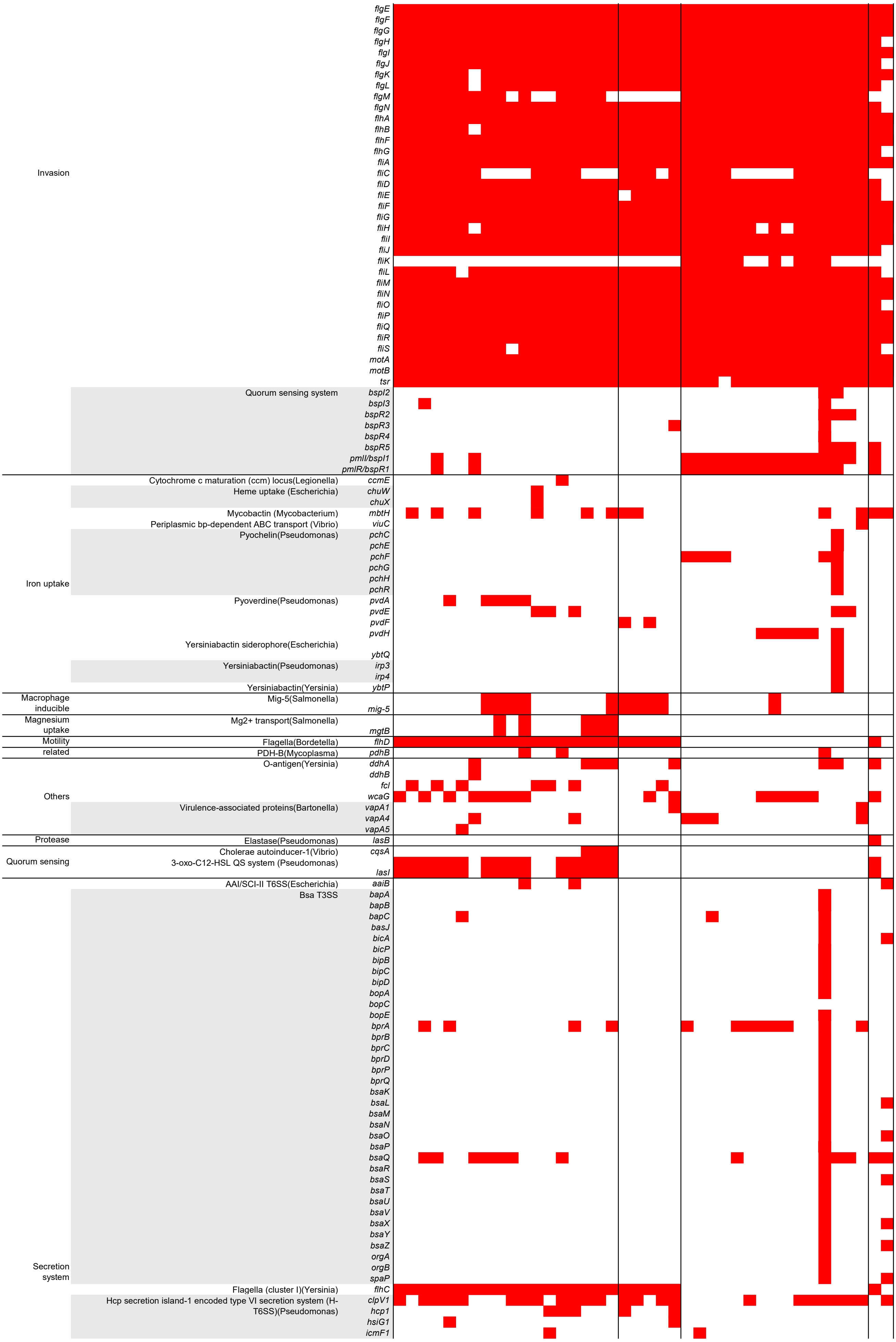

Invasion
flgE
flgF
flgG
flgH
flgI
flgJ
flgK
flgL
flgM
flgN
flhA
flhB
flhF
flhG
fliA
fliC
fliD
fliE
fliF
fliG
fliH
fliI
fliJ
fliK
fliL
fliM
fliN
fliO
fliP
fliQ
fliR
fliS
motA
motB
tsr
Quorum sensing system
bspI2
bspI3
bspR2
bspR3
bspR4
bspR5
pmlI/bspI1
pmlR/bspR1
Iron uptake
Cytochrome c maturation (ccm) locus(Legionella)
ccmE
Heme uptake (Escherichia)
chuW
chuX
Mycobactin (Mycobacterium)
mbtH
Periplasmic bp-dependent ABC transport (Vibrio)
viuC
Pyochelin(Pseudomonas)
pchC
pchE
pchF
pchG
pchH
pchR
Pyoverdine(Pseudomonas)
pvdA
pvdE
pvdF
pvdH
Yersiniabactin siderophore(Escherichia)
ybtQ
Yersiniabactin(Pseudomonas)
irp3
irp4
Yersiniabactin(Yersinia)
ybtP
Macrophage inducible
Mig-5(Salmonella)
mig-5
Magnesium uptake
Mg2+ transport(Salmonella)
mgtB
Motility related
Flagella(Bordetella)
flhD
PDH-B(Mycoplasma)
pdhB
Others
O-antigen(Yersinia)
ddhA
ddhB
fcl
wcaG
Virulence-associated proteins(Bartonella)
vapA1
vapA4
vapA5
Protease
Elastase(Pseudomonas)
lasB
Quorum sensing
Cholerae autoinducer-1(Vibrio)
cqsA
3-oxo-C12-HSL QS system (Pseudomonas)
lasI
Secretion system
AAI/SCI-II T6SS(Escherichia)
aaiB
Bsa T3SS
bapA
bapB
bapC
basJ
bicA
bicP
bipB
bipC
bipD
bopA
bopC
bopE
bprA
bprB
bprC
bprD
bprP
bprQ
bsaK
bsaL
bsaM
bsaN
bsaO
bsaP
bsaQ
bsaR
bsaS
bsaT
bsaU
bsaV
bsaX
bsaY
bsaZ
orgA
orgB
spaP
Flagella (cluster I)(Yersinia)
flhC
Hcp secretion island-1 encoded type VI secretion system (H-T6SS)(Pseudomonas)
clpV1
hcp1
hsiG1
icmF1

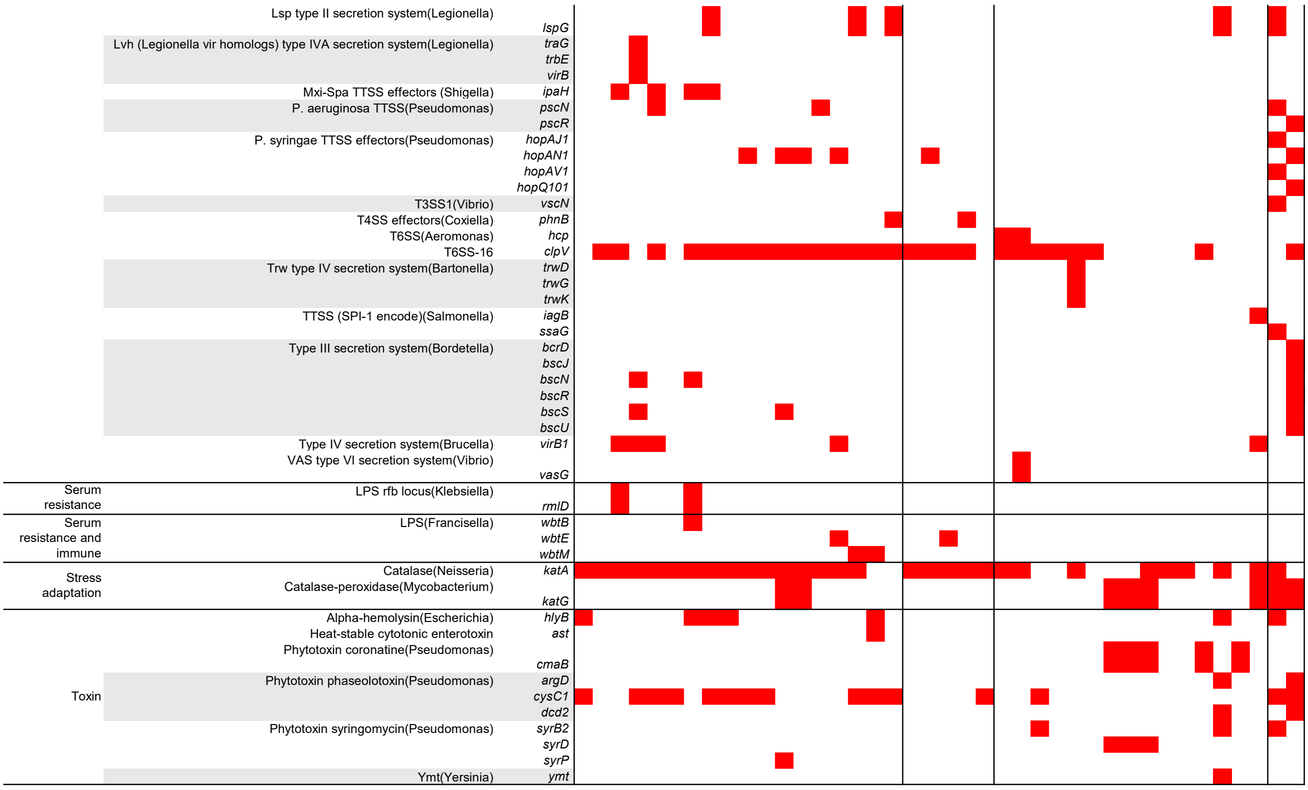
Lsp type II secretion system(Legionella)
lspG
Lvh (Legionella vir homologs) type IVA secretion system(Legionella)
traG
trbE
virB
Mxi-Spa TTSS effectors (Shigella)
ipaH
P. aeruginosa TTSS(Pseudomonas)
pscN
pscR
P. syringae TTSS effectors(Pseudomonas)
hopAJ1
hopAN1
hopAV1
hopQ101
T3SS1(Vibrio)
vscN
T4SS effectors(Coxiella)
phnB
T6SS(Aeromonas)
hcp
T6SS-16
clpV
Trw type IV secretion system(Bartonella)
trwD
trwG
trwK
TTSS (SPI-1 encode)(Salmonella)
iagB
ssaG
Type III secretion system(Bordetella)
bcrD
bscJ
bscN
bscR
bscS
bscU
Type IV secretion system(Brucella)
virB1
VAS type VI secretion system(Vibrio)
vasG
Serum resistance
LPS rfb locus(Klebsiella)
rmlD
Serum resistance and immune
LPS(Francisella)
wbtB
wbtE
wbtM
Stress adaptation
Catalase(Neisseria)
katA
Catalase-peroxidase(Mycobacterium)
katG
Toxin
Alpha-hemolysin(Escherichia)
hlyB
Heat-stable cytotonic enterotoxin
ast
Phytotoxin coronatine(Pseudomonas)
cmaB
Phytotoxin phaseolotoxin(Pseudomonas)
argD
cysC1
dcd2
Phytotoxin syringomycin(Pseudomonas)
syrB2
syrD
syrP
Ymt(Yersinia)
ymt

Fig. S4: VFanalyzer results for the sequenced strains. VF genes identified by VFanalyzer have been listed and their presence in each of the sequenced genomes has been indicated in red. Annotations and classes for each VF gene have been shown, with horizontal lines separating the classes.

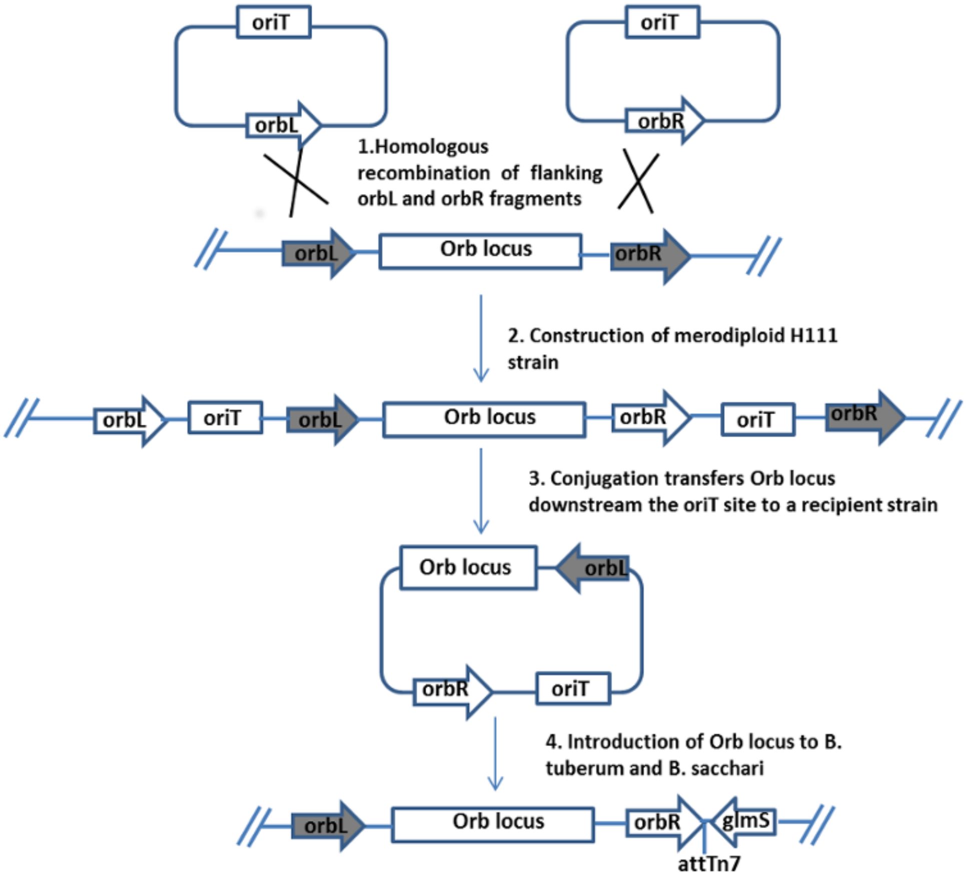

Fig. S5: Ornibactin cluster cloning scheme. (1) *orbL* and *orbR* genes were subcloned into separate miniTn7 capture suicide vectors pFTCAPL and pUCTCAPR respectively. **(2)** Both suicide vectors were homologously recombined into *B. cenocepacia* H111 genome to generate H111 merodiploid strain. **(3)** Conjugative transfer of ornibactin locus starting from the first *oriT* site and terminating at the second *oriT* site into a recipient *E. coli* strain, resulting in a circular pOrnibactin-Tn*7* vector. **(4)** Conjugation of the pOrnibactin-Tn*7* vector with the transposase helper strain pTNS3 into *P. sacchari* and *P. tuberum* resulting in integration of the target *orb* locus into the site specific *att*Tn*7* site

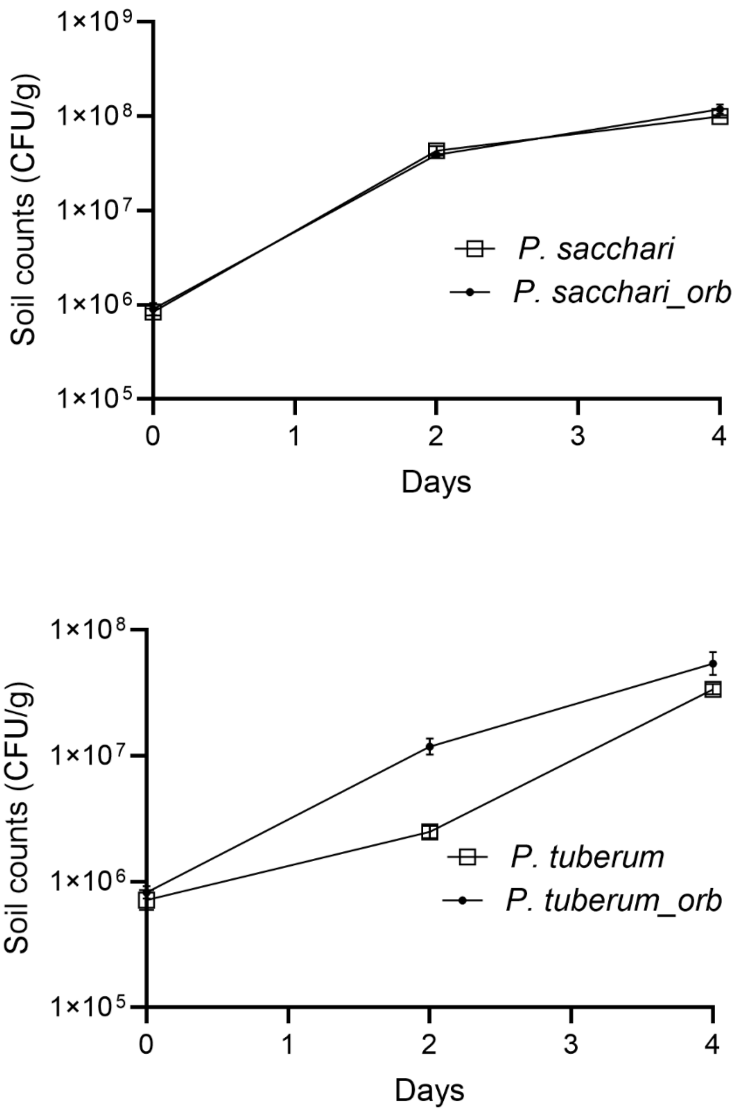

Fig. S6: Growth of *P. sacchari* and *P. tuberum* wt and orb strains in a soil microcosm. The microcosm was inoculated with 6 X 10^8 bacteria per gram of soil and bacterial counts were determined over a period of 4 days. Data represent mean ± S.E of three independent experiments.

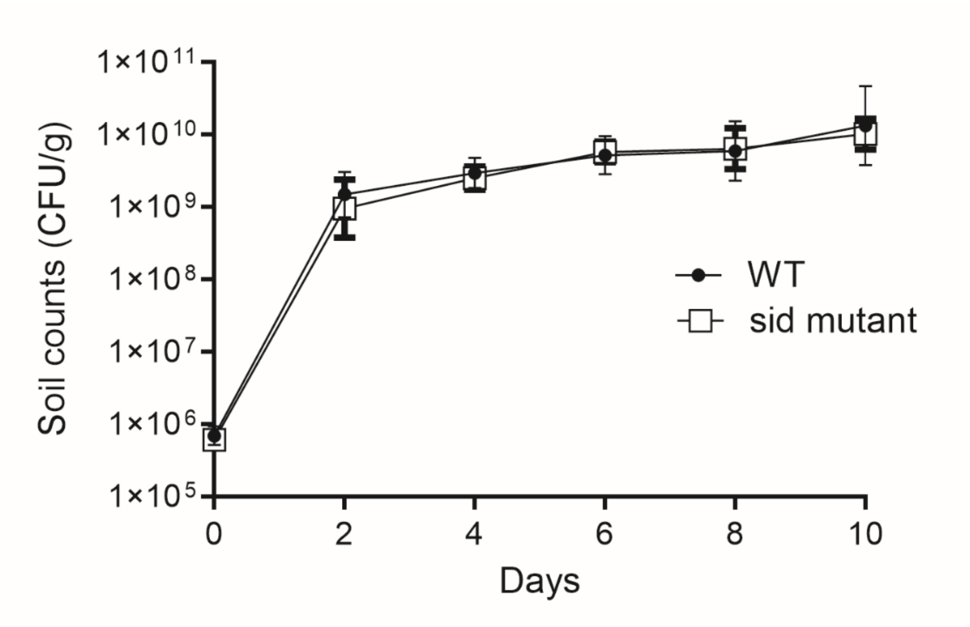

Fig. S7. Siderophores are dispensable for survival of *B. cenocepacia* in a soil microcosm Growth of H111 and siderophore mutant (sid mutant) in a soil microcosm. The microcosm was inoculated with 6 X 10^{8} bacteria per gram of soil and bacterial counts were determined over a period of 10 days. Data represent mean ± S.E of three independent experiments.

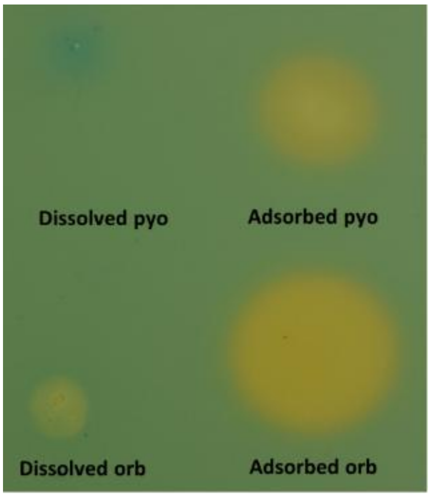

Fig. S8: The majority of ornibactin and pyochelin siderophore molecules were adsorbed onto soil particles in a microcosm. CAS plate displaying dissolved and adsorbed pyochelin (pyo) and ornibactin (orb) fractions from soil microcosms. Dissolved and adsorbed fractions were extracted separately from the soil microcosm after 10 days of inoculation and the fractions were spotted onto CAS plates.

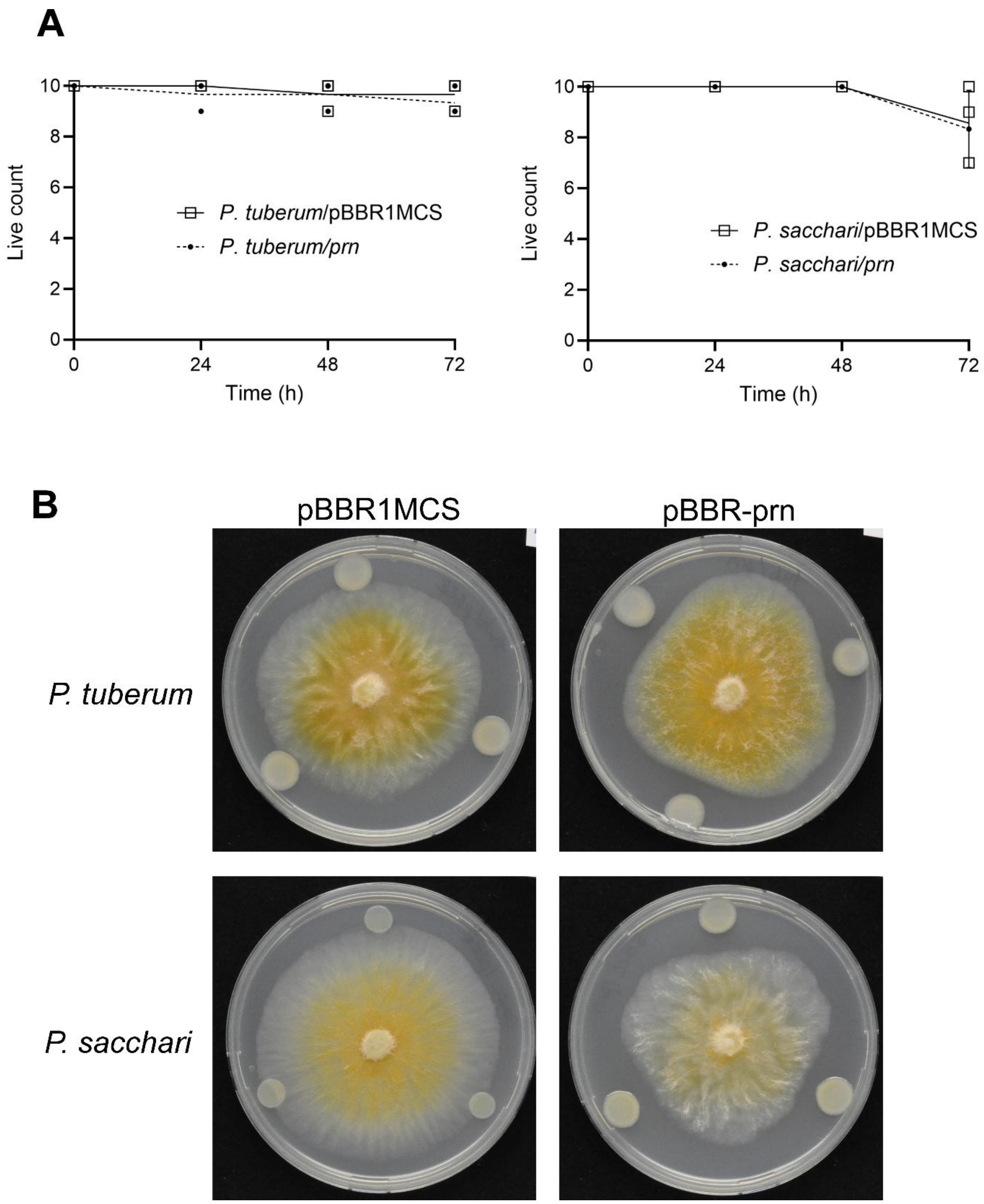

Fig. S9. Transgenic production of pyrrolnitrin confers antifungal activity but does not increase strain pathogenicity. Plasmids pBBR1MCS or pBBR1MCS bearing the *prn* cluster under the control of a constitutive promoter were introduced into either *P. sacchari* or *P. tuberum.* **A.** The resultant strains were assessed for pathogenicity in the wax moth larva model. The assay was carried out in triplicate, n=10 **B.** Antifungal activity against *F. solani.* Shown are representative images from biological triplicate assays.

References

1. Schwyn B, Neilands JB. Universal chemical assay for the detection and determination of siderophores. *Anal Biochem*. 1987;**160**:47-56 https://doi.org/10.1016/0003-2697(87)90612-9
2. Kvitko BH, McMillan IA, Schweizer HP. An improved method for *oriT*-directed cloning and functionalization of large bacterial genomic regions. *Appl Environ Microbiol*. 2013;**79**:4869-78 https://doi.org/10.1128/Aem.00994-13
3. Chain PS, Hernandez-Lucas I, Golding B *et al.* *oriT*-directed cloning of defined large regions from bacterial genomes: identification of the *Sinorhizobium meliloti* pExo megaplasmid replicator region. *J Bacteriol*. 2000;**182**:5486-94 https://doi.org/10.1128/JB.182.19.5486-5494.2000
4. Thomas MS. Iron acquisition mechanisms of the *Burkholderia cepacia* complex. *Biometals*. 2007;**20**:431-52 https://doi.org/10.1007/s10534-006-9065-4
5. Crook MB, Lindsay DP, Biggs MB *et al.* Rhizobial plasmids that cause impaired symbiotic nitrogen fixation and enhanced host invasion. *Mol Plant Microbe Interact*. 2012;**25**:1026-33 https://doi.org/10.1094/MPMI-02-12-0052-R
6. Stephan H, Freund S, Beck W *et al.* Ornibactins-a new family of siderophores from *Pseudomonas*. *Biometals*. 1993;**6**:93-100 https://doi.org/https://doi.org/10.1007/BF00140109
7. Blin K, Shaw S, Kloosterman AM *et al.* antiSMASH 6.0: improving cluster detection and comparison capabilities. *Nucleic Acids Res*. 2021;**49**:29-35 https://doi.org/10.1093/nar/gkab335
8. Blin K, Medema MH, Kazempour D *et al.* antiSMASH 2.0-a versatile platform for genome mining of secondary metabolite producers. *Nucleic Acids Res*. 2013;**41**:W204-12 https://doi.org/10.1093/nar/gkt449
9. Minerdi D, Fani R, Gallo R *et al.* Nitrogen fixation genes in an endosymbiotic *Burkholderia* strain. *Appl Environ Microbiol*. 2001;**67**:725-32 https://doi.org/10.1128/Aem.67.2.725-732.2001
10. Dos Santos PC, Fang Z, Mason SW *et al.* Distribution of nitrogen fixation and nitrogenase-like sequences amongst microbial genomes. *BMC Genomics*. 2012;**13**:162 https://doi.org/10.1186/1471-2164-13-162
11. Mavrodi DV, Peever TL, Mavrodi OV *et al.* Diversity and evolution of the phenazine biosynthesis pathway. *Appl Environ Microbiol*. 2010;**76**:866-79 https://doi.org/10.1128/Aem.02009-09
12. Bian XY, Huang F, Wang HL *et al.* Heterologous production of glidobactins/luminmycins in *Escherichia coli* Nissle containing the glidobactin biosynthetic gene cluster from *Burkholderia* DSM7029. *Chembiochem*. 2014;**15**:2221-24 https://doi.org/10.1002/cbic.201402199
13. Lu SE, Novak J, Austin FW *et al.* Occidiofungin, a unique antifungal glycopeptide produced by a strain of *Burkholderia contaminans*. *Biochemistry*. 2009;**48**:8312-21 https://doi.org/10.1021/bi900814c
14. Kang YW, Carlson R, Tharpe W *et al.* Characterization of genes involved in biosynthesis of a novel antibiotic from *Burkholderia cepacia* BC11 and their role in biological control of Rhizoctonia solani. *Appl Environ Microbiol*. 1998;**64**:3939-47 https://doi.org/10.1128/aem.64.10.3939-3947.1998
15. Mullins AJ, Murray JAH, Bull MJ *et al.* Genome mining identifies cepacin as a plant-protective metabolite of the biopesticidal bacterium *Burkholderia ambifaria*. *Nat Microbiol*. 2019;**4**:996-1005 https://doi.org/10.1038/s41564-019-0383-z
16. Vial L, Lepine F, Milot S *et al.* *Burkholderia pseudomallei, B. thailandensis,* and *B. ambifaria* produce 4-hydroxy-2-alkylquinoline analogues with a methyl group at the 3 position that is required for quorum-sensing regulation. *J Bacteriol*. 2008;**190**:5339-52 https://doi.org/10.1128/Jb.00400-08

17. Moon SS, Kang PM, Park KS *et al.* Plant growth promoting and fungicidal 4-quinolinones from *Pseudomonas cepacia*. *Phytochemistry*. 1996;**42**:365-68 https://doi.org/10.1016/0031-9422(95)00897-7
18. van Pee KH, Ligon JM. Biosynthesis of pyrrolnitrin and other phenylpyrrole derivatives by bacteria. *Nat Prod Rep*. 2000;**17**:157-64 https://doi.org/10.1039/a902138h
19. Hammer PE, Hill DS, Lam ST *et al.* Four genes from *Pseudomonas fluorescens* that encode the biosynthesis of pyrrolnitrin. *Appl Environ Microbiol*. 1997;**63**:2147-54 https://doi.org/10.1128/Aem.63.6.2147-2154.1997
20. Sieber S, Daeppen C, Jenul C *et al.* Biosynthesis and structure-activity relationship investigations of the diazeniumdiolate antifungal agent fragin. *Chembiochem*. 2020;**21**:1587-92 https://doi.org/10.1002/cbic.201900755
21. Florez LV, Scherlach K, Miller IJ *et al.* An antifungal polyketide associated with horizontally acquired genes supports symbiont-mediated defense in *Lagria villosa* beetles. *Nature Communications*. 2018;**9**:2478 https://doi.org/10.1038/s41467-018-04955-6
22. Esmaeel Q, Pupin M, Kieu NP *et al.* *Burkholderia* genome mining for nonribosomal peptide synthetases reveals a great potential for novel siderophores and lipopeptides synthesis. *Microbiologyopen*. 2016;**5**:512-26 https://doi.org/10.1002/mbo3.347
23. Gama S, Hermenau R, Frontauria M *et al.* Iron coordination properties of gramibactin as model for the new class of diazeniumdiolate based siderophores. *Chemistry*. 2021;**27**:2724-33 https://doi.org/10.1002/chem.202003842
24. Hermenau R, Ishida K, Gama S *et al.* Gramibactin is a bacterial siderophore with a diazeniumdiolate ligand system. *Nat Chem Biol*. 2018;**14**:841-43 https://doi.org/10.1038/s41589-018-0101-9
25. Barelmann I MJ-M, Taraz K, Budzikiewicz H. Cepaciachelin, a new catecholate siderophore from *Burkholderia (Pseudomonas) cepacia*. *Z Naturforsch*. 1996;**51c**:627–30 https://doi.org/10.1515/znc-1996-9-1004
26. Jenul C, Sieber S, Daeppen C *et al.* Biosynthesis of fragin is controlled by a novel quorum sensing signal. *Nat Commun*. 2018;**9**:1297 https://doi.org/10.1038/s41467-018-03690-2
27. Alanjary M, Steinke K, Ziemert N. AutoMLST: an automated web server for generating multi-locus species trees highlighting natural product potential. *Nucleic Acids Res*. 2019;**47**:W276-W82 https://doi.org/10.1093/nar/gkz282
28. Pourmohsenin B, Wiese A, Ziemert N. AutoMLST2: a web server for phylogeny and microbial taxonomy. *Nucleic Acids Res*. 2025;**53**:W45-W50 https://doi.org/10.1093/nar/gkaf397
29. Chen LH, Yang J, Yu J *et al.* VFDB: a reference database for bacterial virulence factors. *Nucleic Acids Res*. 2005;**33**:D325-D28 https://doi.org/10.1093/nar/gki008
30. Metsalu T, Vilo J. ClustVis: a web tool for visualizing clustering of multivariate data using Principal Component Analysis and heatmap. *Nucleic Acids Res*. 2015;**43**:W566-70 https://doi.org/10.1093/nar/gkv468
31. Mao D, Bushin LB, Moon K *et al.* Discovery of *scmR* as a global regulator of secondary metabolism and virulence in *Burkholderia thailandensis* E264. *Proc Natl Acad Sci U S A*. 2017;**114**:E2920-E28 https://doi.org/10.1073/pnas.1619529114
32. Wu Y, Seyedsayamdost MR. The polyene natural product thailandamide A inhibits fatty acid biosynthesis in Gram-positive and Gram-negative bacteria. *Biochemistry*. 2018;**57**:4247-51 https://doi.org/10.1021/acs.biochem.8b00678
33. el-Banna N, Winkelmann G. Pyrrolnitrin from *Burkholderia cepacia*: antibiotic activity against fungi and novel activities against *streptomycetes*. *J Appl Microbiol*. 1998;**85**:69-78 https://doi.org/10.1046/j.1365-2672.1998.00473.x
34. Mullins AJ, Mahenthiralingam E. The hidden genomic diversity, specialized metabolite capacity, and revised taxonomy of *Burkholderia sensu lato*. *Front Microbiol*. 2021;**12**:726847 https://doi.org/10.3389/fmicb.2021.726847
35. Webster G, Mullins AJ, Petrova YD *et al.* Polyyne-producing *Burkholderia* suppress *Globisporangium ultimum* damping-off disease of *Pisum sativum* (pea). *Front Microbiol*. 2023;**14**:1240206 https://doi.org/10.3389/fmicb.2023.1240206

36. Petrova YD, Mahenthiralingam E. Discovery, mode of action and secretion of *Burkholderia sensu lato* key antimicrobial specialised metabolites. *Cell Surf*. 2022;**8**:100081 https://doi.org/10.1016/j.tcsw.2022.100081
37. Song LJ, Jenner M, Masschelein J *et al.* Discovery and biosynthesis of gladiolin: a *Burkholderia gladioli* antibiotic with promising activity against *Mycobacterium tuberculosis*. *J Am Chem Soc*. 2017;**139**:7974-81 https://doi.org/10.1021/jacs.7b03382
38. Mahenthiralingam E, Song LJ, Sass A *et al.* Enacyloxins are products of an unusual hybrid modular polyketide synthase encoded by a cryptic *Burkholderia ambifaria* genomic island. *Chemistry and Biology*. 2011;**18**:665-77 https://doi.org/10.1016/j.chembiol.2011.01.020
39. Carr G, Seyedsayamdost MR, Chandler JR *et al.* Sources of diversity in bactobolin biosynthesis by *Burkholderia thailandensis* E264. *Org Lett*. 2011;**13**:3048-51 https://doi.org/10.1021/ol200922s
40. Yamaguchi M, Park HJ, Ishizuka S *et al.* Chemistry and antimicrobial activity of caryoynencins analogs. *J Med Chem*. 1995;**38**:5015-22 https://doi.org/10.1021/jm00026a008
41. Pawar S, Chaudhari A, Prabha R *et al.* Microbial pyrrolnitrin: natural metabolite with immense practical utility. *Biomolecules*. 2019;**9**:443 https://doi.org/10.3390/biom9090443
42. Nisr RB, Russell MA, Chrachri A *et al.* Effects of the microbial secondary metabolites pyrrolnitrin, phenazine and patulin on INS-1 rat pancreatic beta-cells. *FEMS Immunol Med Microbiol*. 2011;**63**:217-27 https://doi.org/10.1111/j.1574-695X.2011.00844.x
43. Schmidt S, Blom JF, Pernthaler J *et al.* Production of the antifungal compound pyrrolnitrin is quorum sensing-regulated in members of the *Burkholderia cepacia* complex. *Environmental microbiology*. 2009;**11**:1422-37 https://doi.org/10.1111/j.1462-2920.2009.01870.x
44. Park J, Lee HH, Jung H *et al.* Transcriptome analysis to understand the effects of the toxoflavin and tropolone produced by phytopathogenic *Burkholderia* on *Escherichia coli*. *J Microbiol*. 2019;**57**:781-94 https://doi.org/10.1007/s12275-019-9330-1
45. Di Santo R, Costi R, Artico M *et al.* Pyrrolnitrin and related pyrroles endowed with antibacterial activities against *Mycobacterium tuberculosis*. *Bioorg Med Chem Lett*. 1998;**8**:2931-6 https://doi.org/10.1016/s0960-894x(98)00526-5
46. Eberl L, Vandamme P. Members of the genus *Burkholderia:* good and bad guys. *F1000Res*. 2016;**5** https://doi.org/10.12688/f1000research.8221.1
47. Zheng WT, Wang X, Zhou HB *et al.* Establishment of recombineering genome editing system in *Paraburkholderia megapolitana* empowers activation of silent biosynthetic gene clusters. *Microb Biotechnol*. 2020;**13**:397-405 https://doi.org/10.1111/1751-7915.13535
48. Vandamme P, Opelt K, Knochel N *et al. Burkholderia bryophila* sp. nov. and *Burkholderia megapolitana* sp. nov., moss-associated species with antifungal and plant-growth-promoting properties. *Int J Syst Evol Microbiol*. 2007;**57**:2228-35 https://doi.org/10.1099/ijs.0.65142-0
49. Elshafie HS, Camele I. An overview of metabolic activity, beneficial and pathogenic aspects of *Burkholderia* spp. *Metabolites*. 2021;**11**:321 https://doi.org/10.3390/metabo11050321
50. Lackner G, Moebius N, Partida-Martinez LP *et al.* Evolution of an endofungal lifestyle: Deductions from the *Burkholderia rhizoxinica* genome. *BMC Genomics*. 2011;**12**:210 https://doi.org/10.1186/1471-2164-12-210
51. Drevinek P, Mahenthiralingam E. *Burkholderia cenocepacia* in cystic fibrosis: epidemiology and molecular mechanisms of virulence. *Clin Microbiol Infect*. 2010;**16**:821-30 https://doi.org/10.1111/j.1469-0691.2010.03237.x
52. Franke J, Ishida K, Hertweck C. Plasticity of the malleobactin pathway and its impact on siderophore action in human pathogenic bacteria. *Chemistry*. 2015;**21**:8010-4 https://doi.org/10.1002/chem.201500757
53. Butt AT, Thomas MS. Iron acquisition mechanisms and their role in the virulence of *Burkholderia* species. *Front Cell Infect Microbiol*. 2017;**7**:460 https://doi.org/10.3389/fcimb.2017.00460
54. Darling P, Chan M, Cox AD *et al.* Siderophore production by cystic fibrosis isolates of *Burkholderia cepacia*. *Infect Immun*. 1998;**66**:874-77 https://doi.org/https://doi.org/10.1128/iai.66.2.874-877.1998

55. Kvitko BH, Goodyear A, Propst KL *et al.* *Burkholderia pseudomallei* known siderophores and hemin uptake are dispensable for lethal murine melioidosis. *Plos Neglect Trop D*. 2012;**6**:e1715 https://doi.org/10.1371/journal.pntd.0001715
56. Schmidt IHE, Gildhorn C, Boning MAL *et al.* *Burkholderia pseudomallei* modulates host iron homeostasis to facilitate iron availability and intracellular survival. *PLoS Negl Trop Dis*. 2018;**12**:e0006096 https://doi.org/10.1371/journal.pntd.0006096
57. Hermenau R, Mehl JL, Ishida K *et al.* Genomics-driven discovery of NO-donating diazeniumdiolate siderophores in diverse plant-associated bacteria. *Angew Chem Int Ed Engl*. 2019;**58**:13024-29 https://doi.org/10.1002/anie.201906326
58. Kooi C, Corbett CR, Sokol PA. Functional analysis of the *Burkholderia cenocepacia* ZmpA metalloprotease. *J Bacteriol*. 2005;**187**:4421-29 https://doi.org/10.1128/JB.187.13.4421-4429.2005
59. Uehlinger S, Schwager S, Bernier SP *et al.* Identification of specific and universal virulence factors in *Burkholderia cenocepacia* strains by using multiple infection hosts. *Infect Immun*. 2009;**77**:4102-10 https://doi.org/10.1128/IAI.00398-09
60. O'Grady EP, Nguyen DT, Weisskopf L *et al.* The *Burkholderia cenocepacia* LysR-type transcriptional regulator ShvR influences expression of quorum-sensing, protease, type II secretion, and *afc* genes. *J Bacteriol*. 2011;**193**:163-76 https://doi.org/10.1128/JB.00852-10
61. Gingues S, Kooi C, Visser MB *et al.* Distribution and expression of the ZmpA metalloprotease in the *Burkholderia cepacia* complex. *J Bacteriol*. 2005;**187**:8247-55 https://doi.org/10.1128/JB.187.24.8247-8255.2005
62. Chin CY, Othman R, Nathan S. The *Burkholderia pseudomallei* serine protease MprA is autoproteolytically activated to produce a highly stable enzyme. *Enzyme Microb Technol*. 2007;**40**:370-77 https://doi.org/10.1016/j.enzmictec.2006.09.014
63. Valade E, Thibault FM, Gauthier YP *et al.* The PmlI-PmlR quorum-sensing system in *Burkholderia pseudomallei* plays a key role in virulence and modulates production of the MprA protease. *J Bacteriol*. 2004;**186**:2288-94 https://doi.org/10.1128/Jb.186.8.2288-2294.2004
64. Herrero M, Delorenzo V, Timmis KN. Transposon vectors containing non-antibiotic resistance selection markers for cloning and stable chromosomal insertion of foreign genes in Gram-negative bacteria. *J Bacteriol*. 1990;**172**:6557-67 https://doi.org/10.1128/jb.172.11.6557-6567.1990.
65. Simon R, Priefer, U., Pühler, A. A broad host range mobilisation system for *in vivo* genetic engineering: transposon mutagenesis in Gram-negative bacteria. *Bio/Technology*. 1983;**1**:784-91 https://doi.org/https://doi.org/10.1038/NBT1183-784
66. Gotschlich A, Huber B, Geisenberger O *et al.* Synthesis of multiple N-acylhomoserine lactones is wide-spread among the members of the *Burkholderia cepacia* complex. *Syst Appl Microbiol*. 2001;**24**:1-14 https://doi.org/10.1078/0723-2020-00013
67. Vanlaere E, Baldwin A, Gevers D *et al.* Taxon K, a complex within the *Burkholderia cepacia* complex, comprises at least two novel species, *Burkholderia contaminans* sp. nov. and *Burkholderia lata* sp. nov. *Int J Syst Evol Microbiol*. 2009;**59**:102-11 https://doi.org/10.1099/ijs.0.001123-0
68. Vandamme P, Goris J, Chen W-M *et al.* *Burkholderia tuberum* sp. nov. and *Burkholderia phymatum* sp. nov., nodulate the roots of tropical legumes. *Syst Appl Microbiol*. 2002;**25**:507-12 https://doi.org/10.1078/07232020260517634
69. Bramer CO, Vandamme P, da Silva LF *et al.* *Burkholderia sacchari* sp. nov., a polyhydroxyalkanoate-accumulating bacterium isolated from soil of a sugar-cane plantation in Brazil. *Int J Syst Evol Microbiol*. 2001;**51**:1709-13 https://doi.org/10.1099/00207713-51-5-1709
70. Carlier A, Burbank L, von Bodman SB. Identification and characterization of three novel EsaI/EsaR quorum-sensing controlled stewartan exopolysaccharide biosynthetic genes in *Pantoea stewartii ssp. stewartii. Mol Microbiol*. 2009;**74**:903-13 https://doi.org/10.1111/j.1365-2958.2009.06906.x

71. Kessler B, de Lorenzo V, Timmis KN. A general system to integrate *lacZ* fusions into the chromosomes of gram-negative eubacteria: regulation of the Pm promoter of the TOL plasmid studied with all controlling elements in monocopy. *Mol Gen Genet*. 1992;**233**:293-301 https://doi.org/10.1007/BF00587591
72. Figurski DH, Helinski DR. Replication of an origin-containing derivative of plasmid RK2 dependent on a plasmid function provided *in trans*. *Proc Natl Acad Sci U S A*. 1979;**76**:1648-52 https://doi.org/10.1073/pnas.76.4.1648
73. O'Grady EP, Viteri DF, Malott RJ *et al.* Reciprocal regulation by the CepIR and CciIR quorum sensing systems in *Burkholderia cenocepacia*. *BMC Genomics*. 2009;**10**:441 https://doi.org/10.1186/1471-2164-10-441
74. Choi KH, Mima T, Casart Y *et al.* Genetic tools for select-agent-compliant manipulation of *Burkholderia pseudomallei*. *Appl Environ Microbiol*. 2008;**74**:1064-75 https://doi.org/10.1128/AEM.02430-07
75. Mannweiler O, Pinto-Carbo M, Lardi M *et al.* Investigation of *Burkholderia cepacia* complex methylomes via single-molecule, real-time sequencing and mutant analysis. *J Bacteriol*. 2021;**203**:e0068320 https://doi.org/10.1128/JB.00683-20
76. Niehs SP, Kumpfmuller J, Dose B *et al.* Insect-associated bacteria assemble the antifungal butenolide gladiofungin by non-canonical polyketide chain termination. *Angew Chem Int Edit*. 2020;**59**:23122-26 https://doi.org/10.1002/anie.202005711
77. Greenberg EP, Chandler JR, Seyedsayamdost MR. The chemistry and biology of bactobolin: a 10-year collaboration with natural product chemist extraordinaire Jon Clardy. *J Nat Prod*. 2020;**83**:738-43 https://doi.org/10.1021/acs.jnatprod.9b01237
